# Sample size variation in single-time post-dose assessment *vs* multi-time post-dose assessment

**DOI:** 10.12688/f1000research.124917.1

**Published:** 2022-12-21

**Authors:** Sarfaraz Sayyed, Ashwini Mathur, Asha Kamath

**Affiliations:** 1 Analytics, Novartis Healthcare Pvt. Ltd., Hyderabad, Telangana, 500080, India; 2 Clinical Technology & Innovation, Novartis Ireland Ltd., Dublin, Ireland; 3 Department of Data Science, Manipal academy of higher studies, Manipal, karnataka, 576104, India

**Keywords:** sample size estimation; longitudinal study; repeated measure analysis; univariate analysis

## Abstract

**Background:** Many randomized trials measure a continuous outcome simultaneously at baseline and after taking the drug. For a single continuous post-treatment outcome, the sample size calculation is simple, but if there are assessments at multiple time points post-treatment then this longitudinal data may give more insights by analyzing the data using the repeated measures method. Also, if the sample size is calculated using the single time-point method for longitudinal data, it may lead to a larger than required sample size, increasing the cost and time.

**Methods:** In this research, an effort is made to determine the size of the sample for repeated measures case and then compared with the single post-baseline case. The sample sizes were examined under different scenarios for the continuous type of response variable. Under Mean contrast and Diff contrast the sample sizes were calculated with different correlations. These two scenarios were again examined under compound symmetry as well as Auto regressive of order 1 type of correlation structure in longitudinal data. The graphical presentation is given for better visualization of the scenarios.

**Results:** Sample size required for highly correlated longitudinal data using multi timepoint sample size derivation method led to much smaller sample size requirement as compared to single timepoint sample size calculation method.

**Conclusions:** This study will help researchers to make better decisions in choosing the right method for sample size determination which may reduce the time and cost of carrying out the experiment. Also, we must carefully assess which method to go with when the correlation is weak. More complex correlation structures are not studied in this article but can be studied in the same fashion.

## Introduction

‘Sample size’ is a term used in any experiment for defining the number of subjects selected or observed in the experiment. Sample used in an experiment is a subset of the entire target population. The target population refers to a group of individuals among whom the research is intended to be conducted in order to draw conclusions about the population. For e.g., for a type II diabetic test drug all humans who are suffering from type II diabetes would form the target population.

The number of participants in the sample effects the precision of our estimate and the power of the study to draw conclusions. The probability with which one would reject null hypothesis when null hypothesis is not true is said to be the power of the statistical test. So, power gives an idea on the chances of not committing an error of Type II. The size of the sample and the effect size are major factors which affects the power of a study. These are key concepts in designing a clinical trial. ^1^ Sample size calculation is an important activity in setting up clinical trials to ensure achievement of trial objectives.

As an illustration, consider a study to compare the performance of a professional athlete taking a particular protein shake *versus* athletes who do not consume any special protein shakes. Narrowing down attention to a portion of the wider group is essential to enable tracking of the eating habits of every elite athlete in the world. This would entail choosing 100 professional athletes for our study at random; in this case, 100 would be the sample size. Based on the data gathered from a sample of 100 elite athletes, the study’s findings potentially characterize the population of all athletes in the sports industry. Lack of full coverage of the target population would result in the outcomes of the study having a margin of error. Given the limitation to examine every situation, the absolute precision of the effect of protein shake on the athlete’s performance would be hard to measure. Sampling error ^2^ is the term used to describe this level of uncertainty or inaccuracy. It affects the estimator’s precision, which is a metric that is important for the chosen target population of all professional athletes.

Although sampling error cannot be completely eliminated, it can be reduced. A larger sample typically has a narrower margin of error. We require an appropriate sample size to examine and contrast in order to provide an accurate picture of the effects of protein shake consumption on performance. Note that there comes a point where increasing the sample size has no further effect on the sampling error (law of diminishing returns). ^3^

## Background

Longitudinal studies take a longer time but help in determining causality as well as monitoring the trend over time. To see how sample size calculation was addressed in published longitudinal studies we searched the databases such as Scopus, Web of Science, PubMed, ScienceDirect, and Google Scholar using a range of key terms: “designing clinical trials”, “sample size calculation”, “longitudinal studies”, “randomized trials” and “repeated measures”. The ensuing literature review did not reveal much information on details of how the sample size was calculated for these published longitudinal studies. ^4^^,^^5^

Formulae for deriving sample size in longitudinal studies is available from several papers. ^6^^,^^7^ Basagaña, Liao and Spiegelma ^8^ published a study in which the power as well as the sample size are discussed for time-varying exposures, but how this is practically applied to a longitudinal study design and its outcome is undocumented in published papers. Pourhoseingholi *et al.,* ^9^ and Karimollah ^10^ both published about the importance of various components for calculating the sample size in medical studies or clinical trials where often there would be more than one post baseline assessment, but sample size calculation is shown assuming single post baseline assessment. Manja and Lakshminrusimha published a two-part study ^11^^,^^12^ which does give a good explanation on clinical research design, but sample size is not discussed in detail.

Most of the published studies which have assessments at multiple time points calculate the sample size based on the change from study end time point to baseline whereas a smaller number of papers emphasize on the use of multiple time points into consideration for calculating the sample size. ^6^^–^^8^

The need for this research was prompted by this lack of proper usage of sample size calculation for longitudinal studies and to further explore which method for sample size calculation should be used in a longitudinal study resulting in correlated outcome data.

## Objective

To explore the variation in sample size by considering multiple time point assessment versus the change from baseline to a single endpoint.

### Notation and framework

In an experiment for testing certain hypothesis with parallel group design, two or more independent groups are considered to be treated under different scenarios in order to compare the outcome of the scenarios. In our study we would consider the objective of comparison of two drugs.

Let $Y_{\mathrm{ij}}$ (X _ij_) be the outcome of interest at *j* ^th^ (j = 1, 2, 3, …, *t*) time point for the *i* ^th^ ( *i* = 1, 2, 3, …, *n*) patient in the two groups.

For the parallel group design, these 2 *n* patients will be divided into two groups with 1: 1 ratio where one arm is assigned to receive the test drug and the other arm is assigned to receive the comparator drug.

Let $\mu_{1}\&\mu_{2}$ be the population mean change from baseline for the test drug and comparator drug respectively.

Let $\bar{Y}\&\bar{X}$ be the sample mean change from baseline outcome for the test drug and comparator drug respectively, where $\bar{Y}∼N\left(\mu_{1},\frac{\sigma^{2}}{n}\right)$ and $\bar{X}∼N\left(\mu_{2},\frac{\sigma^{2}}{n}\right)$ .

## Methods

### Method for Sample size with single time point assessment analysis

*Change at single post-baseline assessment from baseline (Single time assessment analysis)*

In a parallel group design study with two arms of equal size let the hypothesis be set as:

$H_{0}:\mu_{1}-\mu_{2}=\delta =0$ , There is no difference between the effects of test and comparator drug.

Vs

$H_{a}:\mu_{1}-\mu_{2}=\delta >0$ , Test drug is having larger effect as compared to comparator.

The test statistic assuming common standard deviation σ for both arms will be given by

$$T=\frac{\bar{Y}-\bar{X}}{\sqrt{\;\mathrm{Var}\left(\bar{Y}-\bar{X}\right)}}=\frac{\bar{Y}-\bar{X}}{\sqrt{\;\frac{\sigma^{2}}{n}+\frac{\sigma^{2}}{n}}}=\frac{\bar{Y}-\bar{X}}{\sqrt{\;\frac{2\sigma^{2}}{n}}}$$ (1)

Now If $H_{0}$ is true (and $\mu_{1}=\mu_{2}),$ then $T∼N\left(0,1\right)$ , else if $H_{a}$ is true (i.e., δ>0) , then T will still be a normal but with a mean greater than zero.

If Type II error is denoted by β then power will be simply 1−β and power is the probability to reject $H_{0}$ when $H_{a}$ is true. In probability equation it could be written as

$$\mathrm{Pr}\left[\text{Reject}H_{0}|H_{a}\;\text{is true}\right]=\mathrm{Pr}\left[T>z_{1-\alpha }\right|\delta >0]=1-\beta ,$$ (2)

where $z_{1-\alpha }$ is the threshold or the critical value which is $\left(1-\alpha \right)$ quantile from Gaussian distribution and α is the type I error or the level of significance.

$$T∼N\left(\frac{\delta }{\sqrt{\;\frac{2\sigma^{2}}{n}}},1\right),\text{Under}\;H_{a}\to T-\frac{\delta }{\sqrt{\;\frac{2\sigma^{2}}{n}}}=Z∼N\left(0,1\right)$$ (3)

$$\begin{array}{c} \text{Power function}=\mathrm{Pr}\left[T>z_{1-\alpha }\right|\delta >0]=\mathrm{Pr}\left\{\frac{\;\bar{Y}-\bar{X}-\delta }{\sqrt{\frac{2\sigma^{2}}{n}}}>z_{1-\alpha }-\frac{\delta }{\sqrt{\frac{2\sigma^{2}}{n}}}|\delta >0\right\}\\ =\mathrm{Pr}\left\{Z>z_{1-\alpha }-\frac{\delta }{\sqrt{\frac{2\sigma^{2}}{n}}}|\delta >0\right\}\\ =1-\Phi \left(z_{1-\alpha }-\sqrt{2n}\left(\frac{\delta }{2\sigma }\right)\right),\text{where}\;\Phi \left(z\right)=\mathrm{Pr}\left(Z\leq z\right) \end{array}$$ (4)

Now in any study we would be looking for

$$\begin{array}{c} 1-\Phi \left(z_{1-\alpha }-\sqrt{\frac{n}{2}}\left(\frac{\delta }{\sigma }\right)\right)\geq 1-\beta \\ \to \beta \geq \Phi \left(z_{1-\alpha }-\sqrt{\frac{n}{2}}\left(\frac{\delta }{\sigma }\right)\right)\;\text{or}\;z_{\beta }\geq z_{1-\alpha }-\sqrt{\frac{n}{2}}\left(\frac{\delta }{\sigma }\right) \end{array}$$ (5)

After solving equation 5, we get

$$n\geq \frac{2{\left(z_{1-\alpha }+z_{1-\beta }\right)}^{2}\sigma^{2}\;}{{\left(\mu_{1}-\mu_{2}\right)}^{2}}=\frac{2{\left(z_{\alpha }+z_{\beta }\right)}^{2}\;}{{\left[\left(\mu_{1}-\mu_{2}\right)/\sigma \right]}^{2}}$$ (6)

Here *n* is the sample size required per arm. We will use these formulae in calculating the sample size for single post baseline time point analysis.

### Method for Sample size with multiple time point assessment analysis

*Change from baseline with post baseline assessment at multiple timepoints (Multiple time points analysis)*

In a parallel group design study with two arms of equal size and assessments taken at multiple time points let the hypothesis be set as:

$H_{a}:\psi_{c}=0$ , there is no difference between the effects of test and comparator drug.

$H_{a}:\psi_{c}>0$ , test drug is having larger effect as compared to comparator.

Where $\psi_{c}=\mathrm{C\Lambda}$ ,

Let $C=\left[\begin{array}{c} c_{1}\\ c_{2}\\ \vdots \\ c_{i}\\ \vdots \\ c_{t} \end{array}\right]$ be the contrast to be tested for the hypothesis and let $\Lambda =\left[\begin{array}{c} \mu_{11}-\mu_{21}\\ \mu_{12}-\mu_{22}\\ \vdots \\ \mu_{1i}-\mu_{2i}\\ \vdots \\ \mu_{1t}-\mu_{2t} \end{array}\right]$

$\mu_{1i}\&\mu_{2i}$ are the mean effect in arm one and arm two at time point “ *i*” respectively in a study with t time points. $c_{i}$ can take any value depending on the hypothesis we want to test.

For e.g., if we want to see the difference between two drugs for change from baseline when *t*=2, then $c_{1}=-1\;\text{and}\;c_{2}=1$ and the resulting $\psi_{c}$ will be

$$\begin{array}{c} \psi_{c}=\mathrm{C\Lambda}=\left[-1\;1\right].\left[\begin{array}{c} \mu_{11}-\mu_{21}\\ \mu_{12}-\mu_{22} \end{array}\right]\\ \;=-\left(\mu_{11}-\mu_{21}\right)+\mu_{12}-\mu_{22}\\ \;=\left(\mu_{12}–\mu_{11}\right)-\left(\mu_{22}-\mu_{21}\right)\\ \;=\text{change in Drug}\;1-\text{change in Drug}\;2. \end{array}$$ (7)

$$\text{Common Variance covariance matrix}=\left[\begin{array}{cccc} \sigma_{1}^{2} & \sigma_{12} & \cdots & \sigma_{1t}\\ \sigma_{12} & \sigma_{2}^{2} & \ddots & \sigma_{2t}\\ \vdots & \vdots & \ddots & \vdots \\ \sigma_{i1} & \sigma_{i2} & \ddots & \sigma_{it}\\ \vdots & \vdots & \ddots & \vdots \\ \sigma_{1t} & \sigma_{2t} & \cdots & \sigma_{t}^{2} \end{array}\right]$$

Where $\sigma_{i}^{2}$ is the variance at time point i and $\sigma_{\mathrm{ij}}$ represents the covariance between time point *i* and *j*.

The test statistic assuming similar variance-covariance matrix for both arms will be given by

$$T=\frac{\psi_{c}}{\sqrt{\;\mathrm{Var}\left(\psi_{c}\right)}}=\frac{\psi_{c}}{\sqrt{\mathrm{Var}\left(\mathrm{C\Lambda}\right)\;}}=\frac{\psi_{c}}{\sqrt{C^{\prime }.\mathrm{Var}\left(\Lambda \right).C\;}}$$ (8)

Consider the $\mathrm{Var}\left(\Lambda \right)$ ,

$$\mathrm{Var}\left(\Lambda \right)=\mathrm{Var}\left[\begin{array}{c} \mu_{11}-\mu_{21}\\ \mu_{12}-\mu_{22}\\ \vdots \\ \mu_{1i}-\mu_{2i}\\ \vdots \\ \mu_{1t}-\mu_{2t} \end{array}\right]=\mathrm{Var}\left[\begin{array}{c} \delta_{1}\\ \delta_{2}\\ \vdots \\ \delta_{i}\\ \vdots \\ \delta_{t} \end{array}\right]=\left[\begin{array}{cccc} \frac{2}{n}.\sigma_{1}^{2} & \frac{4}{n}.\sigma_{12} & \cdots & \frac{4}{n}.\sigma_{1t}\\ \frac{4}{n}.\sigma_{12} & \frac{2}{n}.\sigma_{2}^{2} & \ddots & \frac{4}{n}.\sigma_{2t}\\ \vdots & \vdots & \ddots & \vdots \\ \frac{4}{n}.\sigma_{i1} & \frac{4}{n}.\sigma_{i2} & \ddots & \frac{4}{n}.\sigma_{it}\\ \vdots & \vdots & \ddots & \vdots \\ \frac{4}{n}.\sigma_{1t} & \frac{4}{n}.\sigma_{2t} & \cdots & \frac{2}{n}.\sigma_{t}^{2} \end{array}\right]$$

$$\mathrm{Var}\left(\Lambda \right)=\frac{2}{n}.\left[\begin{array}{cccc} \sigma_{1}^{2} & 2\sigma_{12} & \cdots & 2\sigma_{1t}\\ \sigma_{12} & \sigma_{2}^{2} & \ddots & \sigma_{2t}\\ \vdots & \vdots & \ddots & \vdots \\ \sigma_{i1} & 2\sigma_{i2} & \ddots & 2\sigma_{it}\\ \vdots & \vdots & \ddots & \vdots \\ 2\sigma_{1t} & 2\sigma_{2t} & \cdots & \sigma_{t}^{2} \end{array}\right]$$ (9)

Solving for $C^{\prime }.\mathrm{Var}\left(\Lambda \right).C\;$ , we get

$$\begin{array}{c} C^{\prime }.\mathrm{Var}\left(\Lambda \right).C=\left[\begin{array}{c} c_{1}\;c_{2}\ldots c_{i}\ldots c_{t} \end{array}\right].\frac{2}{n}.\left[\begin{array}{cccc} \sigma_{1}^{2} & 2\sigma_{12} & \cdots & 2\sigma_{1t}\\ \vdots & \vdots & \ddots & \vdots \\ \sigma_{i1} & 2\sigma_{i2} & \ddots & 2\sigma_{\mathrm{it}}\\ \vdots & \vdots & \ddots & \vdots \\ 2\sigma_{1t} & 2\sigma_{2t} & \cdots & \sigma_{t}^{2} \end{array}\right]\left[\begin{array}{c} c_{1}\\ c_{2}\\ \vdots \\ c_{i}\\ \vdots \\ c_{t} \end{array}\right]\\ \;=\frac{2}{n}.\left[\sum_{i=1}^{n}c_{i}^{2}\sigma_{i}^{2}+2\sum_{i<j}^{n}c_{i}c_{j}\sigma_{\mathrm{ij}}\right]=\frac{2}{n}.\sigma_{c}^{2},\\ \text{where}\;\sigma_{c}^{2}=\left[\sum_{i=1}^{n}c_{i}^{2}\sigma_{i}^{2}+2\sum_{i<j}^{n}c_{i}c_{j}\sigma_{\mathrm{ij}}\right]\; \end{array}$$ (10)

Solving equations Equation 8 and Equation 9 for *T* we get

$$\begin{array}{c} T=\frac{\psi_{c}}{\sqrt{C^{\prime }.\mathrm{Var}\left(\Lambda \right).C\;}}=\frac{\psi_{c}}{\sqrt{\frac{2}{n}.\left[\sum_{i=1}^{n}c_{i}^{2}\sigma_{i}^{2}+2\sum_{i<j}^{n}c_{i}c_{j}\sigma_{\mathrm{ij}}\right]\;}}\\ =\sqrt{\frac{n}{2}}\;\left(\frac{\psi_{c}}{\sqrt{\sigma_{c}^{2}\;}}\right) \end{array}$$ (11)

Now, If we follow similar steps as we did in single time point analysis above, we get

$$\beta \geq \Phi \left(z_{1-\alpha }-\sqrt{\frac{n}{2}}\;\left(\frac{\psi_{c}}{\sqrt{\sigma_{c}^{2}\;}}\right)\right)\;\text{or}\;z_{\beta }\geq z_{1-\alpha }-\sqrt{\frac{n}{2}}\;\left(\frac{\psi_{c}}{\sqrt{\sigma_{c}^{2}\;}}\right)$$ (12)

And solving Equation 12, we get ^13^

$$\begin{array}{c} n\geq \frac{2{\left(z_{\alpha }+z_{\beta }\right)}^{2}\sigma_{c}^{2}\;}{\psi_{c}^{2}}\;\\ \text{with}\\ \psi_{c}=\sum_{i=1}^{t}c_{i}\left(\mu_{1i}-\mu_{2i}\right)\;\text{and}\;\sigma_{c}^{2}=\sum_{i=1}^{t}c_{i}^{2}\sigma_{i}^{2}+2\sum_{i<j}^{t}c_{i}c_{j}\sigma_{\mathrm{ij}}\; \end{array}$$ (13)

$\sigma_{i}^{2}=\text{common variance in the}\;\mathrm{two}\;\text{groups}\;\mathrm{at}\;\text{timepoint}\;i.$

$\sigma_{ij}=\text{common covariance in the}\;\mathrm{two}\;\text{groups between timepoint}\;i\;\text{and}\;j.$

$c_{i}=\text{contrast applied}\;\mathrm{at}\;\text{timepoint}\;i\;\text{and}\;t\;\text{represents the number of time points}.$

We will use the formulae specified in Equation 13 to calculate the sample size for multiple time point analysis.

### Calculation of sample size

Appropriate sample size was calculated for multi-time and single time cases with different scenarios to achieve an overall mean treatment difference (0.9 points) between two treatment groups with common standard deviation (SD) (3.6 points) allowing 5% two-sided type I error and 85% power. The effect size and standard deviation used here are based on a real study. ^14^ This was a three-year study with primary endpoint assessment at end of each year but the sample size calculation in this study was done on the basis of single time point. Since this study failed to recruit the expected number of patients and also had lot of missing data, the characteristics for the primary endpoints from the second year were used as it had equal number of patients in both arms and stabilized assessments.

### Sample size (single time point case)

We considered a two-arm parallel group scenario with one baseline and one post baseline timepoints to assess on change from baseline in absolute scale. Using the formulae in Equation 6 above for single timepoint analysis the sample size required per arm was 287 cases to show statistical significance.

### Sample size (longitudinal case)

Here again we considered two arm parallel groups with multiple timepoints and for studying we investigated six cases i.e., three, four, five, six, eight, and 10 timepoints. Each of this case corresponds to number of assessments including baseline. Three timepoints corresponded to the case with one baseline and two post baseline assessments, four timepoints corresponded to the case with one baseline and three post baseline assessments, five timepoints corresponded to the case with one baseline and four post baseline assessments and so on.

Figure 1 and Figure 2 represents each of this case as a line in the plot under different contrast types and correlation structures.

**Figure 1.**
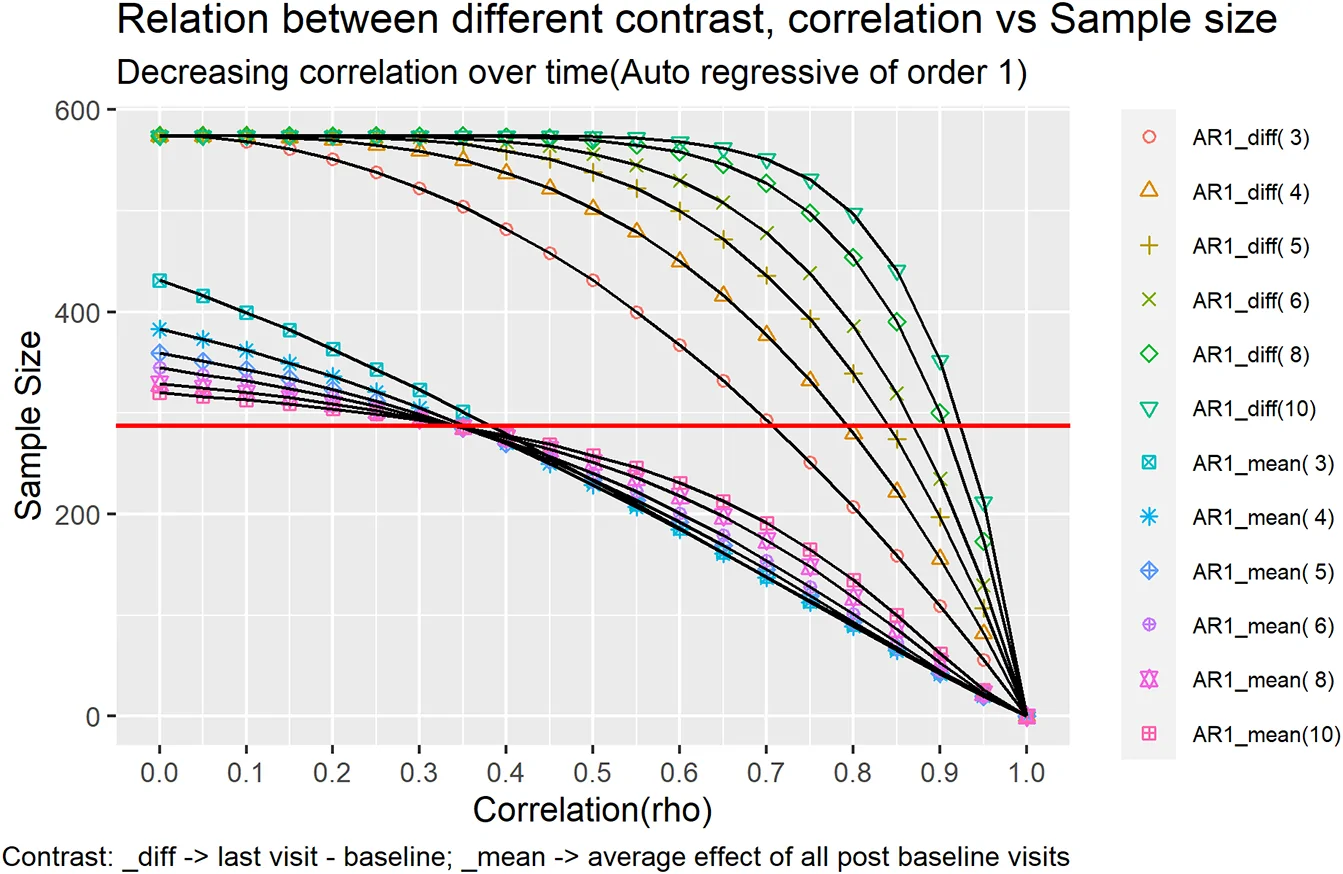
Simulation results with compound symmetry correlation structure.

**Figure 2.**
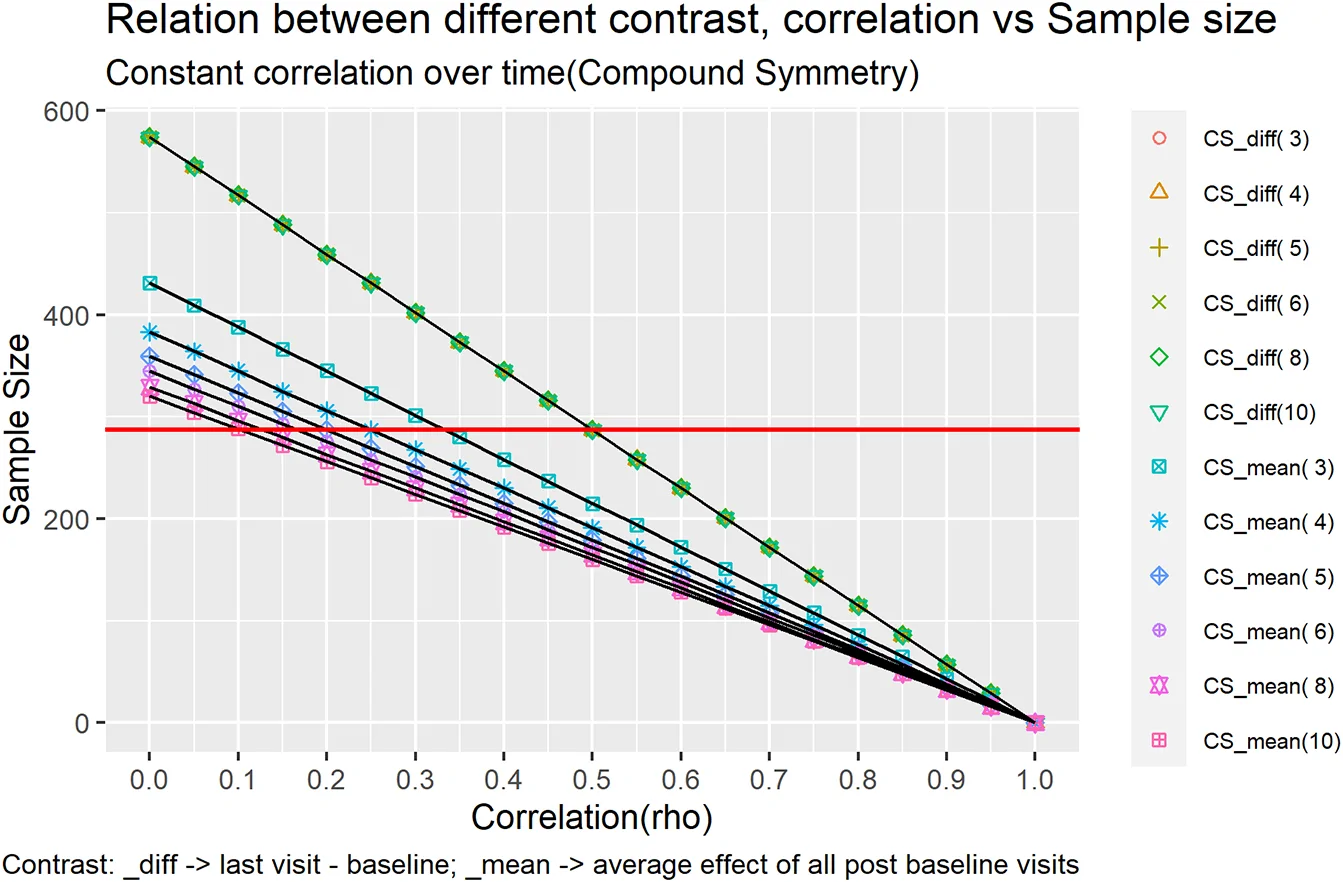
Simulation results with auto regressive order 1 correlation structure.

Keeping the SD as 3.6 we tried to vary over two different correlation structures:

Compound symmetry (CS)

Compound Symmetry just means that all the variances are equal and all the covariances are equal. So, the same variance and covariance are used for all subjects. In compound symmetry the covariances across the subjects and the variances (pooled within the group) of the different repeated measures are homogeneous.

$$\left[\begin{array}{ccccc} \sigma^{2} & \sigma^{2}\rho & \sigma^{2}\rho & \cdots & \sigma^{2}\rho \\ \sigma^{2}\rho & \sigma^{2} & \sigma^{2}\rho & \cdots & \sigma^{2}\rho \\ \sigma^{2}\rho & \sigma^{2}\rho & \sigma^{2} & \cdots & \sigma^{2}\rho \\ \vdots & \ddots & \vdots & \vdots & \vdots \\ \sigma^{2}\rho & \sigma^{2}\rho & \sigma^{2}\rho & \cdots & \sigma^{2} \end{array}\right]$$

Where *σ* ^2^ is the common variance assumed to be similar over time and ρ is the assumed correlation. Order of variance covariance matrix will be t × t . ‘ *t*’ is the number of time points.

Auto regressive of order 1(AR1)

This is the homogeneous variance first-order autoregressive structure. Any two elements that are adjacent have a correlation that is equal to rho ( *ρ*), those separated by a third will have correlation *ρ* ^2^, and so on. rho is restricted such that –1< *ρ* <1.

$$\left[\begin{array}{ccccc} \sigma^{2} & \sigma^{2}\rho & \sigma^{2}\rho^{2} & \cdots & \sigma^{2}\rho^{t-1}\\ \sigma^{2}\rho & \sigma^{2} & \sigma^{2}\rho & \cdots & \sigma^{2}\rho^{t-2}\\ \sigma^{2}\rho^{2} & \sigma^{2}\rho & \sigma^{2} & \cdots & \sigma^{2}\rho^{t-3}\\ \vdots & \ddots & \vdots & \vdots & \vdots \\ \sigma^{2}\rho^{t-1} & \sigma^{2}\rho^{t-2} & \sigma^{2}\rho^{t-3} & \cdots & \sigma^{2} \end{array}\right]$$

Where *σ* ^2^ is the common variance assumed to be similar over time and *ρ* is the assumed correlation. Order of variance covariance matrix will be t x t . ‘ *t*’ is the number of time points.

Also, we considered different scenarios of how we want to analyze the results at the end as different contrasts as described below.

### Contrast for repeated measures

We tried to investigate two types of contrasts.

1. Time-related contrasts i.e., mean over time (mean contrast).

**Rationale:** This contrast comes from the chronic types of disease where regular medicine is required, and the effect remains only for some time and then the disease condition reverses back.
  This will be labelled in the legend of Figure 1 as CS_mean( *i*) and in the legend of Figure 2 as AR1_mean( *i*). For e.g., for five timepoints the contrast would look like *c*(-1, ¼, ¼, ¼, ¼).
2. Mean Difference (change at last time point from baseline) (diff contrast).

**Rationale:** This contrast comes from the types of disease where each dose reduces the disease severity and the over the course it is totally removed from the patient’s body. Here the total effect at the end of the treatment course as compared to the baseline is of interest.
  This will be labelled in the legend of Figure 1 as CS_diff( *i*) and in the legend of Figure 2 as AR1_diff( *i*). For e.g., for five timepoints the contrast would look like *c*(-1, 0, 0, 0, 1).

Sample size was calculated for correlation ranging from 0 – 1 with intervals of 0.05 for both the plots Figure 1 and Figure 2. Sample Size was derived using the formulae mentioned in Equation 13.

### CS variance structure with ‘mean over time’ and ‘mean difference’ contrasts

The red horizontal line represents the sample size from single time point assessment approach.

CS_diff( *i*), CS_mean( *i*) – *i* represents the no. of visits used for sample size calculation, *i* = 3,4,5,6,8,10.

### AR(1) covariance structure with ‘mean over time’ and ‘mean difference’ contrasts

The red horizontal line represents the sample size from single time point assessment approach.

AR1_diff( *i*), AR1_mean( *i*) – *i* represents the no. of visits used for sample size calculation, *i* = 3,4,5,6,8,10.

## Results

### Under CS type of variance covariance structure ( Figure 1)

All the trend lines for mean difference type of contrast overlaps each other. For mean difference type of contrast, the sample size doesn’t change for an increase/decrease in the number of visits. It changes with the correlation i.e., highly correlated (rho > 0.5) timepoints would need less sample size as compared to low correlated timepoints. Also, for correlation = 0.5 the multiple assessment sample size coincides with that of single time point assessment.

However, the sample size does vary when the contrast is set to mean over time. Multiple time point assessment with more timepoints requires less sample size as compared to that of multiple time point assessment with less time points for e.g., the multiple time point assessment with three timepoint requires 86 per arm with correlation 0.8 and the multiple time point assessment with 10 timepoints requires 64 per arm. On the same lines the multiple time point assessment with three timepoints requires 258 per arm with correlation 0.4 and the multiple time point assessment with 10 timepoints requires 192 per arm. This trend shows that the sample size required reduces when the correlation increases. For correlation = 0.1 to 0.35 the sample size coincides with that of single time point assessment.

### Under AR(1) type of variance covariance structure ( Figure 2)

Under mean over time contrast the multiple time point assessment requires lower sample size as compared to single time point assessment (287 per arm) for correlation greater than 0.35 and the sample size increases as correlation goes below 0.35.

Whereas for mean difference contrast the multiple time point assessment requires lower sample size as compared to single time point assessment (287 per arm) for correlation greater than 0.7 but requires higher sample size for correlation less than 0.7.

The trend changes shape for **mean difference** contrast vs mean over time contrast. Also, at certain point the increase in sample size attenuates for e.g., in case of mean difference type contrast with 10 time points the sample size required doesn’t changes when correlation drops below 0.55.

## Discussion

One of the hurdles in considering the longitudinal methodology for sample size calculation is the assumption on the covariance matrix. It is often easy to estimate the variance of single timepoint as compared to estimating the variance-covariance matrix for multiple time points.

The above derivations were done for trial design with parallel group, 1:1 ratio and two arms. If the ratio changes or if we have more than two arms or if the design is crossover, then the effective overall sample size would get effected in both the cases i.e., sample size with single time point as well as sample size with multiple timepoints, but the trend would remain the same as shown above in the figures and the results will still hold good. Similar trends should hold for other variance – covariance structures though they have not been simulated here.

## Conclusion

Sample size changes depending on the analysis type and the data collected. Both the graphs in Figure 1 and Figure 2 in this study reveal that if response is assessed at multiple timepoints and the correlation between the paired observations is high (> 0.6) then one should consider using repeated measures analysis and consequently determine the size of the sample that is based on the multiple time points scenario which results in lower sample size requirement as compared to the sample size derived assuming single timepoint response assessment. This would reduce the cost, resources, and time in conducting the experiment fastening the new drug development. Also, repeated measures analyses will not drop the patients in which they have certain missing data as compared to single point analysis where the patient will be dropped if the response is missing hence may help in retaining the power.

Sample size derivation using longitudinal design method for studies with multiple assessments can be considered of substantial benefit in cost and time although the challenge of estimating the variance-covariance matrix remains.

## Software availability

Software available from: The Comprehensive R Archive Network ( https://cran.r-project.org/)

Source code available from: https://github.com/Sarfaraz-Sayyed/Sample-Size-Variation.

Archived source code at time of publication: https://zenodo.org/badge/latestdoi/547747570. ^15^

License: MIT License.

## Data Availability

No data are associated with this article.
