# Sample size variation in single-time post-dose assessment *vs* multi-time post-dose assessment

**DOI:** 10.12688/f1000research.124917.3

**Published:** 2026-02-05

**Authors:** Sarfaraz Sayyed, Ashwini Mathur, Asha Kamath

**Affiliations:** 1 Analytics, Novartis Healthcare Pvt. Ltd., Hyderabad, Telangana, 500080, India; 2 Clinical Technology & Innovation, Novartis Ireland Ltd., Dublin, Ireland; 3 Department of Data Science, Manipal Academy of Higher Studies, Manipal, Karnataka, 576104, India

**Keywords:** sample size estimation; longitudinal study; repeated measure analysis; univariate analysis

## Abstract

**Background:**

Many randomized trials measure a continuous outcome simultaneously at baseline and after taking the drug. For a single continuous post-treatment outcome, the sample size calculation is simple, but if there are assessments at multiple time point post-treatment then this longitudinal data may give more insights by analyzing the data using the repeated measures method. Also, if the sample size is calculated using the single time-point method for longitudinal data, it may lead to a larger than required sample size, increasing the cost and time.

**Methods:**

In this research, an effort is made to determine the size of the sample for repeated measures case and then compare with the single post-baseline case. The sample sizes were examined under different scenarios for the continuous type of response variable. Under ‘Mean contrast’ and ‘Diff contrast’ the sample sizes were calculated with different correlations. These two scenarios were again examined under compound symmetry as well as discrete Auto regressive of order 1 type of correlation structure in longitudinal data. The graphical presentation is given for better visualization of the scenarios.

**Results:**

The sample size required for highly correlated longitudinal data using the multi-timepoint sample size derivation method led to a smaller sample size requirement compared to the single timepoint sample size calculation method.

**Conclusions:**

This study will help researchers to make better decisions in choosing the right method for sample size determination which may reduce the time and cost of conducting the experiment. Additionally, it is crucial to carefully evaluate and choose the appropriate method when the correlation is weak, as this can significantly impact the accuracy of the results. More complex correlation structures are not studied in this article but can be studied in the same fashion.

## Introduction

Understanding the concept of “sample size” is crucial for anyone involved in scientific research or clinical trials. The sample size refers to the number of subjects selected or observed in an experiment. This sample is a subset of the entire target population, which includes all individuals relevant to the study. For instance, in a study evaluating a new drug for type II diabetes, the target population would consist of all individuals suffering from this condition.

The sample size significantly influences the precision of our estimates and the study’s power, which is probability of correctly rejecting the null hypothesis when it is false. Essentially, higher power means a greater chance of detecting a true effect.

Two primary factors impact the power of a study: the sample size and the effect size. A larger sample size generally increases the study’s power, enhancing our ability to draw accurate conclusions. Effect size, on the other hand, measures the magnitude of the difference or relationship being studied between the groups.

In clinical trials, carefully calculating the sample size is essential. Correctly calculated sample size ensures that the study is adequately powered to meet its objectives, providing reliable and meaningful results. This meticulous planning is fundamental to advancing medical knowledge and improving patient care.

By paying close attention to sample size, researchers can design robust experiments that yield trustworthy insights, contributing to scientific progress and better health outcomes.

As an illustration, consider a study to compare the performance of a professional athlete taking a particular protein shake *versus* athletes who do not consume any special protein shakes. Narrowing down attention to a portion of the wider group is essential to enable tracking of the eating habits of every elite athlete in the world. Suppose this entails choosing one hundred professional athletes for our study at random; in this case, one hundred would be the sample size. Based on the data gathered from a sample of one hundred elite athletes, the study’s findings potentially characterize the population of all athletes in the sports industry. Lack of full coverage of the target population would result in the study’s outcome having a margin of error. Sampling error ^1^ refers to the random variation between a sample estimate and the true population parameter due to observing only a subset of the population. It reduces the precision of an estimator (e.g., widening confidence intervals), which is crucial for generalizing results to the target population (e.g., all professional athletes). Greater precision enhances confidence in results, enabling more accurate analysis and informed decision-making.

Although sampling error cannot be completely eliminated, it can be reduced. ^2^ A larger sample typically has a narrower margin of error. We require an appropriate sample size to examine and provide an accurate picture of the effects of protein shake consumption on performance. Note that increasing the sample size will help in reducing the sampling error but it does not address the non-sampling errors.

## Background

Longitudinal studies typically require longer follow-up periods but offer important advantages, including the assessment of temporal trends and stronger support for causal inference. While numerous longitudinal studies are available in literature, the purpose of this section is not to provide a comprehensive review of such research. Rather, it aims to illustrate that even in well-established and prestigious journals, longitudinal studies often provide limited detail regarding sample size calculation.

To examine how sample size calculations are reported in applied longitudinal studies, we conducted a targeted search of major bibliographic databases, including ‘Scopus’, ‘Web of Science’, ‘PubMed’, ‘ScienceDirect’, and ‘Google Scholar’ using a range of key terms: “designing clinical trials”, “sample size calculation”, “longitudinal studies”, “randomized trials” and “repeated measures”. This targeted review indicated that detailed descriptions of sample size calculation were frequently limited or absent in the reported methods of published longitudinal studies, as illustrated by selected examples. ^3^^,^^4^ In contrast, methodological papers providing statistical formulae and frameworks for sample size determination in longitudinal designs are well documented in the literature. ^5^^,^^6^ Additional examples from applied studies, ^7^^–^^11^ discussed in the following paragraph, further highlight this recurring shortfall in reporting practice. ^1^^–^^3^

Basagana, Liao and Spiegelman ^7^ published a study in which the power as well as the sample size are discussed for time-varying exposures, but how this is practically applied to a longitudinal study design and its outcome is undocumented in published papers. Pourhoseingholi et al., ^8^ and Karimollah ^9^ both published about the importance of various components for calculating the sample size in medical studies or clinical trials where often there would be more than one post baseline assessment, but sample size calculation is shown assuming single post baseline assessment. Manja and Lakshminrusimha published a two-part study ^10^^,^^11^ which does give a good explanation on clinical research design, but sample size is not discussed in detail.

Most of the published studies which have assessments at multiple time points calculate the sample size based on the change from study end time point to baseline whereas a smaller number of papers emphasize on the use of multiple time points into consideration for calculating the sample size. ^5^^–^^7^

The need for this research was prompted by this lack of proper usage of sample size calculation for longitudinal studies and to further explore which method for sample size calculation should be used in a longitudinal study resulting in correlated outcome data.

A wide range of methodologies and software tools for sample size and power calculation in longitudinal studies already exist ^12^^,^^13^ (e.g., Diggle et al.; Ahn et al.; [R package references]). The present work does not seek to replace these approaches, but rather to complement them by explicitly contrasting single-time-point and multiple-time-point designs under simple and commonly assumed correlation structures.

## Objective

To explore the variation in sample size by considering multiple time point assessment versus the change from baseline to a single endpoint.

### Notation and framework

Consider an experiment for testing certain hypothesis with parallel group design, having two independent groups to be treated under different scenarios to compare the outcome between the two groups. In our study we would consider the objective of comparison of two drugs.

Let $Y_{\mathrm{ij}}$ ( $X_{\mathrm{ij}}$ ) be the outcome of interest at $j^{\mathrm{th}}$ ( *j* = 1, 2, 3, … , *t*) time point for the $i^{\mathrm{th}}$ ( *i* *=* 1, 2, 3, … , *n*) patient in the two groups.

For the parallel group design, these 2 *n* patients will be divided into two groups with 1: 1 ratio where one arm is assigned to receive the test drug, and the other arm is assigned to receive the comparator drug.

Let $\mu_{1}$ & $\mu_{2}$ be the population mean for the test drug and comparator drug, respectively.

Let $\bar{Y}$ & $\bar{X}$ be the sample mean outcome for the test drug and comparator drug respectively. The distribution of $\bar{Y}$ & $\bar{X}$ can be approximated as $N\left(\mu_{1},\frac{\sigma^{2}}{n}\right)$ and $N\left(\mu_{2},\frac{\sigma^{2}}{n}\right)$ respectively under common standard deviation assumption. Under standard regularity conditions, the sampling distribution of the sample mean $\bar{X}$ is approximately normal, with the accuracy of this approximation improving as the sample size increases, in accordance with the central limit theorem. ^14^

## Methods

### Method for sample size with single time point assessment analysis

*Change at single post-baseline assessment (single time assessment analysis)*

In a parallel group design study with two arms of equal size let the hypothesis be set as:

$H_{0}:\mu_{1}=\mu_{2}$ , no difference between the effects of test drug and comparator drug.

Vs

$H_{a}:\mu_{1}>\mu_{2}$ , test drug effect is greater than comparator and $\mu_{1}-\mu_{2}=$ *δ, δ >* 0 is the expected difference.

The test statistic assuming known common standard deviation ‘ σ ’ (estimated from previous clinical trial data with same molecule which could be phase1, phase2 trials for same indication or pivotal trials with same molecule for different indication) for both arms will be given by

$$T=\frac{\bar{Y}-\bar{X}}{\sqrt{\;\mathrm{Var}\left(\bar{Y}-\bar{X}\right)}}=\frac{\bar{Y}-\bar{X}}{\sqrt{\;\frac{\sigma^{2}}{n}+\frac{\sigma^{2}}{n}}}=\frac{\bar{Y}-\bar{X}}{\sqrt{\;\frac{2\sigma^{2}}{n}}}$$ (1)

Now if $H_{0}$ is true ( $\mu_{1}=\mu_{2}$ ), then T∼N(0,1) , else if $H_{a}$ is true (i.e., $\mu_{1}>\mu_{2}$ ) , then *T* will still follow Gaussian distribution but with a mean greater than zero.

If Type II error is denoted by β then power will be simply 1−β and power is the probability to reject $H_{0}$ when $H_{a}$ is true. In probability equation it could be written as

$$\mathrm{Pr}\left[\text{Reject}H_{0}|H_{a}\;\text{is true}\right]=\mathrm{Pr}\left[T>z_{1-\alpha }\right|\delta ]=1-\beta ,$$ (2)

where $z_{1-\alpha }$ is the threshold or the critical value which is $\left(1-\alpha \right)$ quantile from Gaussian distribution and α is the type I error or the level of significance.

$$T∼N\left(\frac{\delta }{\sqrt{\;\frac{2\sigma^{2}}{n}}},1\right),\text{under}\;H_{a}\to T-\frac{\delta }{\sqrt{\;\frac{2\sigma^{2}}{n}}}=Z∼N\left(0,1\right)$$ (3)

$$\begin{array}{c} \text{Power function}=\mathrm{Pr}\left[T>z_{1-\alpha }\right|\delta ]=\mathrm{Pr}\left\{\frac{\;\bar{Y}-\bar{X}-\delta }{\sqrt{\frac{2\sigma^{2}}{n}}}>z_{1-\alpha }-\frac{\delta }{\sqrt{\frac{2\sigma^{2}}{n}}}\right\}\\ \;=1-\Phi \left(z_{1-\alpha }-\sqrt{2n}\left(\frac{\delta }{2\sigma }\right)\right),\text{where}\;\Phi \left(z\right)=\mathrm{Pr}\left(Z\leq z\right) \end{array}$$ (4)

Now in any study we would be looking for an inequality as below.

$$\begin{array}{c} 1-\Phi \left(z_{1-\alpha }-\sqrt{\frac{n}{2}}\left(\frac{\delta }{\sigma }\right)\right)\geq 1-\beta \\ \to \beta \geq \Phi \left(z_{1-\alpha }-\sqrt{\frac{n}{2}}\left(\frac{\delta }{\sigma }\right)\right)\;\text{or}\;z_{\beta }\geq z_{1-\alpha }-\sqrt{\frac{n}{2}}\left(\frac{\delta }{\sigma }\right) \end{array}$$ (5)

Upon solving Equation 5, we get ^15^

$$n\geq \frac{2{\left(z_{1-\alpha }+z_{1-\beta }\right)}^{2}\sigma^{2}\;}{{\left(\bar{Y}-\bar{X}\right)}^{2}}=\frac{2{\left(z_{\alpha }+z_{\beta }\right)}^{2}\;}{{\left[\left(\bar{Y}-\bar{X}\right)/\sigma \right]}^{2}}$$ (6)

Here, *n* is the sample size required per arm. We will use these formula in calculating the sample size for single post baseline time point analysis.

### Method for sample size with multiple time points assessment analysis

*Post baseline assessment at multiple timepoints (multiple time points analysis)*

In a parallel group design study with two arms of equal size and assessments taken at ‘ *t*’ time points,

Let $C=\left[\begin{array}{c} c_{1}\\ c_{2}\\ \vdots \\ c_{i}\\ \vdots \\ c_{t} \end{array}\right]$ be the contrast to be tested for the hypothesis and let $\Lambda =\left[\begin{array}{c} \mu_{11}-\mu_{21}\\ \mu_{12}-\mu_{22}\\ \vdots \\ \mu_{1i}-\mu_{2i}\\ \vdots \\ \mu_{1t}-\mu_{2t} \end{array}\right]$

With the hypothesis to be tested as:

$H_{0}:\psi_{c}=0$ , there is no difference between the effects of test and comparator drug.

$H_{a}:\psi_{c}>0$ , test drug is having larger effect as compared to comparator.

Where $\psi_{c}=C^{\prime }\Lambda \;\text{and}$ $C^{\prime }$ is the transpose of C.

$\mu_{1i}$ & $\mu_{2i}$ are the mean effect in arm one and arm two at time point ‘ *i*’ respectively in a study with ‘ *t*’ time points. $c_{i}$ can take any value depending on the hypothesis we want to evaluate.

For example, if we want to see the difference between two drugs when *t* = 2, then $c_{1}=-1\;\text{and}\;c_{2}=1$ and the resulting $\psi_{c}$ will be

$$\begin{array}{c} \psi_{c}=C^{\prime }\Lambda =\left[-1\;1\right].\left[\begin{array}{c} \mu_{11}-\mu_{21}\\ \mu_{12}-\mu_{22} \end{array}\right]\\ \;=-\left(\mu_{11}-\mu_{21}\right)+\mu_{12}-\mu_{22}\\ \;=\left(\mu_{12}–\mu_{11}\right)-\left(\mu_{22}-\mu_{21}\right)\\ \;=\text{effect in Drug}\;1-\text{effect in Drug}\;2. \end{array}$$ (7)

The variance-covariance matrix (estimated from previous clinical trial data with same molecule which could be phase1, phase2 trials for same indication or pivotal trials with same molecule for different indication), assumed to be known and similar between the two arms is given by

$$\left[\begin{array}{cccc} \sigma_{1}^{2} & \sigma_{12} & \cdots & \sigma_{1t}\\ \sigma_{21} & \sigma_{2}^{2} & \ddots & \sigma_{2t}\\ \vdots & \vdots & \ddots & \vdots \\ \sigma_{i1} & \sigma_{i2} & \ddots & \sigma_{\mathrm{it}}\\ \vdots & \vdots & \ddots & \vdots \\ \sigma_{t1} & \sigma_{t2} & \cdots & \sigma_{t}^{2} \end{array}\right]$$

Where $\sigma_{i}^{2}$ is the variance at time point ‘ *i*’ and $\sigma_{\mathrm{ij}}$ represents the covariance between time point ‘ *i*’ and ‘ *j*’.

The test statistic is given by

$$T=\frac{{\hat{\psi }}_{c}}{\sqrt{\;\mathrm{Var}\left({\hat{\psi }}_{c}\right)}}=\frac{{\hat{\psi }}_{c}}{\sqrt{\mathrm{Var}\left(C^{\prime }\hat{\Lambda }\right)\;}}=\frac{{\hat{\psi }}_{c}}{\sqrt{C^{\prime }.\mathrm{Var}\left(\hat{\Lambda }\right).C\;}}$$ (8)

Where, ${\hat{\psi }}_{c}$ and $\hat{\Lambda }\;$ denote the sample-based estimators of the corresponding population quantities, such that the resulting expression constitutes a valid test statistic such that

$${\hat{\psi }}_{c}=C^{\prime }\hat{\Lambda }\;\text{and}\;\hat{\Lambda }=\left[\begin{array}{c} {\bar{Y}}_{1}-{\bar{X}}_{1}\\ {\bar{Y}}_{2}-{\bar{X}}_{2}\\ \vdots \\ {\bar{Y}}_{i}-{\bar{X}}_{i}\\ \vdots \\ {\bar{Y}}_{t}-{\bar{X}}_{t} \end{array}\right]$$

with ${\bar{Y}}_{i}$ & ${\bar{X}}_{i}$ being the sample mean at time ‘ *i*’ in both the arms respectively.

Consider the $\mathrm{Var}\left(\hat{\Lambda }\right)$ ,

$$\mathrm{Var}\left(\hat{\Lambda }\right)=\mathrm{Var}\left[\begin{array}{c} {\bar{Y}}_{1}-{\bar{X}}_{1}\\ {\bar{Y}}_{2}-{\bar{X}}_{2}\\ \vdots \\ {\bar{Y}}_{i}-{\bar{X}}_{i}\\ \vdots \\ {\bar{Y}}_{t}-{\bar{X}}_{t} \end{array}\right]=\left[\begin{array}{cccc} \frac{2}{n}.\sigma_{1}^{2} & \frac{2}{n}.\sigma_{12} & \cdots & \frac{2}{n}.\sigma_{1t}\\ \frac{2}{n}.\sigma_{21} & \frac{2}{n}.\sigma_{2}^{2} & \ddots & \frac{2}{n}.\sigma_{2t}\\ \vdots & \vdots & \ddots & \vdots \\ \frac{2}{n}.\sigma_{i1} & \frac{2}{n}.\sigma_{i2} & \ddots & \frac{2}{n}.\sigma_{\mathrm{it}}\\ \vdots & \vdots & \ddots & \vdots \\ \frac{2}{n}.\sigma_{t1} & \frac{2}{n}.\sigma_{t2} & \cdots & \frac{2}{n}.\sigma_{t}^{2} \end{array}\right]=\frac{2}{n}.\left[\begin{array}{cccc} \sigma_{1}^{2} & \sigma_{12} & \cdots & \sigma_{1t}\\ \sigma_{21} & \sigma_{2}^{2} & \ddots & \sigma_{2t}\\ \vdots & \vdots & \ddots & \vdots \\ \sigma_{i1} & \sigma_{i2} & \ddots & \sigma_{\mathrm{it}}\\ \vdots & \vdots & \ddots & \vdots \\ \sigma_{t1} & \sigma_{t2} & \cdots & \sigma_{t}^{2} \end{array}\right]$$ (9)

Solving for $C^{\prime }.\mathrm{Var}\left(\hat{\Lambda }\right).C$ using Equation 9, we get

$$C^{\prime }.\mathrm{Var}\left(\hat{\Lambda }\right).C=\left[\begin{array}{c} c_{1}\;c_{2}\;\ldots c_{i}\ldots c_{t} \end{array}\right].\frac{2}{n}.\left[\begin{array}{cccc} \sigma_{1}^{2} & \sigma_{12} & \cdots & \sigma_{1t}\\ \sigma_{21} & \sigma_{2}^{2} & \ddots & \sigma_{2t}\\ \vdots & \vdots & \ddots & \vdots \\ \sigma_{i1} & \sigma_{i2} & \ddots & \sigma_{\mathrm{it}}\\ \vdots & \vdots & \ddots & \vdots \\ \sigma_{t1} & \sigma_{t2} & \cdots & \sigma_{t}^{2} \end{array}\right].\left[\begin{array}{c} c_{1}\\ c_{2}\\ \vdots \\ c_{i}\\ \vdots \\ c_{t} \end{array}\right]=\frac{2}{n}.\left[\sum_{i=1}^{t}c_{i}^{2}\sigma_{i}^{2}+2\sum_{i<j}^{t}c_{i}c_{j}\sigma_{\mathrm{ij}}\right]=\frac{2}{n}.\sigma_{c}^{2},\;\text{where}\;\sigma_{c}^{2}=\;\left[\sum_{i=1}^{t}c_{i}^{2}\sigma_{i}^{2}+2\sum_{i<j}^{t}c_{i}c_{j}\sigma_{\mathrm{ij}}\right]$$ (10)

Using Equation 10 in Equation 8 for *T* we get

$$T=\frac{{\hat{\psi }}_{c}}{\sqrt{C^{\prime }.\mathrm{Var}\left(\hat{\Lambda }\right).C\;}}=\frac{{\hat{\psi }}_{c}}{\sqrt{\frac{2}{n}.\left[\sum_{i=1}^{n}c_{i}^{2}\sigma_{i}^{2}+2\sum_{i<j}^{n}c_{i}c_{j}\sigma_{\mathrm{ij}}\right]\;}}=\sqrt{\frac{n}{2}}\;\left(\frac{{\hat{\psi }}_{c}}{\sqrt{\sigma_{c}^{2}\;}}\right)$$ (11)

Now, if we follow similar steps as we did in single time point analysis above, we get the following inequality.

$$\beta \geq \Phi \left(z_{1-\alpha }-\sqrt{\frac{n}{2}}\;\left(\frac{{\hat{\psi }}_{c}}{\sqrt{\sigma_{c}^{2}\;}}\right)\right)\;\text{or}\;z_{\beta }\geq z_{1-\alpha }-\sqrt{\frac{n}{2}}\;\left(\frac{{\hat{\psi }}_{c}}{\sqrt{\sigma_{c}^{2}\;}}\right)$$ (12)

And solving Equation 12, we get ^12^

$$\begin{array}{c} n\geq \frac{2{\left(z_{\alpha }+z_{\beta }\right)}^{2}\sigma_{c}^{2}\;}{{\hat{\psi }}_{c}^{2}}\;\\ \text{with}\\ {\hat{\psi }}_{c}=\sum_{i=1}^{t}c_{i}\left({\bar{Y}}_{i}-{\bar{X}}_{i}\right)\;\text{and}\;\sigma_{c}^{2}=\left[\sum_{i=1}^{t}c_{i}^{2}\sigma_{i}^{2}+2\sum_{i<j}^{t}c_{i}c_{j}\sigma_{\mathrm{ij}}\right] \end{array}$$ (13)

$\sigma_{i}^{2}$ = common variance(assumed known)in the two groups at timepoint ‘ *i*’

$\sigma_{ij}$ = common covariance(assumed known) in the two groups between timepoint ‘ *i*’ and ‘ *j*’.

$c_{i}$ = contrast applied at timepoint ‘ *i*’ and ‘ *t*’ represents the number of time points.

We will use the formulae specified in Equation 13 to calculate the sample size for multiple time point analysis.

### Calculation of sample size

Consider a 3-year study with assessments at baseline, 3 months, 6 months, 9 months, 12 months, 15 months, 18 months, 24 months, 30 months, 36 months. Appropriate sample size was calculated for multi-timepoint and single timepoint cases with different scenarios to achieve a difference of 0.9 points at 36 months between two treatment groups with an increasing trend from baseline. The different scenarios considered were with respect to equally spaced time points. Below table shows the timepoints selected for each scenario under longitudinal data sample size calculation. For single timepoint assessment only the baseline and 36 months were used.

**Table 1.** Scenarios with timepoints selected.

Scenarios	Visits considered
3 timepoints	Baseline, 18m, 36m
4 timepoints	Baseline, 12m, 24m, 36m
5 timepoints	Baseline, 9m, 18m, 24m, 36m
6 timepoints	Baseline, 6m, 15m, 24m, 30m, 36m
8 timepoints	Baseline, 6m, 9m, 12m, 15m, 18m, 24m, 36m
10 timepoints	Baseline, 3m, 6m, 9m, 12m, 15m, 18m, 24m, 30m, 36m

‘m’ - months.

A common standard deviation (SD) of 3.6 points was used; with type I error rate chosen as 5% and the power set at 85%. The effect size and standard deviation used here are observed in a real study. ^16^ This was a three-year study with primary endpoint assessment at the end of year 3, but the sample size calculation in this study was done based on single time point. Since this study failed to recruit the expected number of patients and had a lot of missing data, the characteristics up until the second year’s evaluation were utilized because they featured a balanced number of patients in both treatment groups and had consistent measurements.

### Sample size (single time point case)

We considered a two-arm parallel group scenario with assessment at baseline and 36 months to assess the change from baseline in absolute scale. Using the formulae in Equation 6 above for single timepoint analysis to test the hypothesis, $H_{0}:\mu_{1}=\mu_{2}$ vs $H_{a}:\mu_{1}>\mu_{2}\;(\text{where}\;\mu_{1}-\mu_{2}=0.9,$ which is the expected difference), the sample size required per arm was 287 cases to achieve a difference of 0.9 points at the end of 36 months between two treatment groups with a common standard deviation (SD) of 3.6 points choosing type I error rate as 5% and the power set at 85% to show statistical significance.

### Sample size (longitudinal case)

Here again we considered two arm parallel groups with multiple timepoints and for studying we investigated six cases i.e., three, four, five, six, eight, and ten timepoints. Each of these cases correspond to multiple assessments including baseline. Three timepoints corresponded to the case with one baseline and two post baseline assessments, four timepoints corresponded to the case with one baseline and three post baseline assessments, five timepoints corresponded to the case with one baseline and four post baseline assessments, similarly other cases corresponded to one visit of baseline and remaining visits from pot baseline assessments.

Figure 1 and Figure 2 represents each of these cases as a line in the plot under different contrast types and correlation structures.

**Figure 1.**
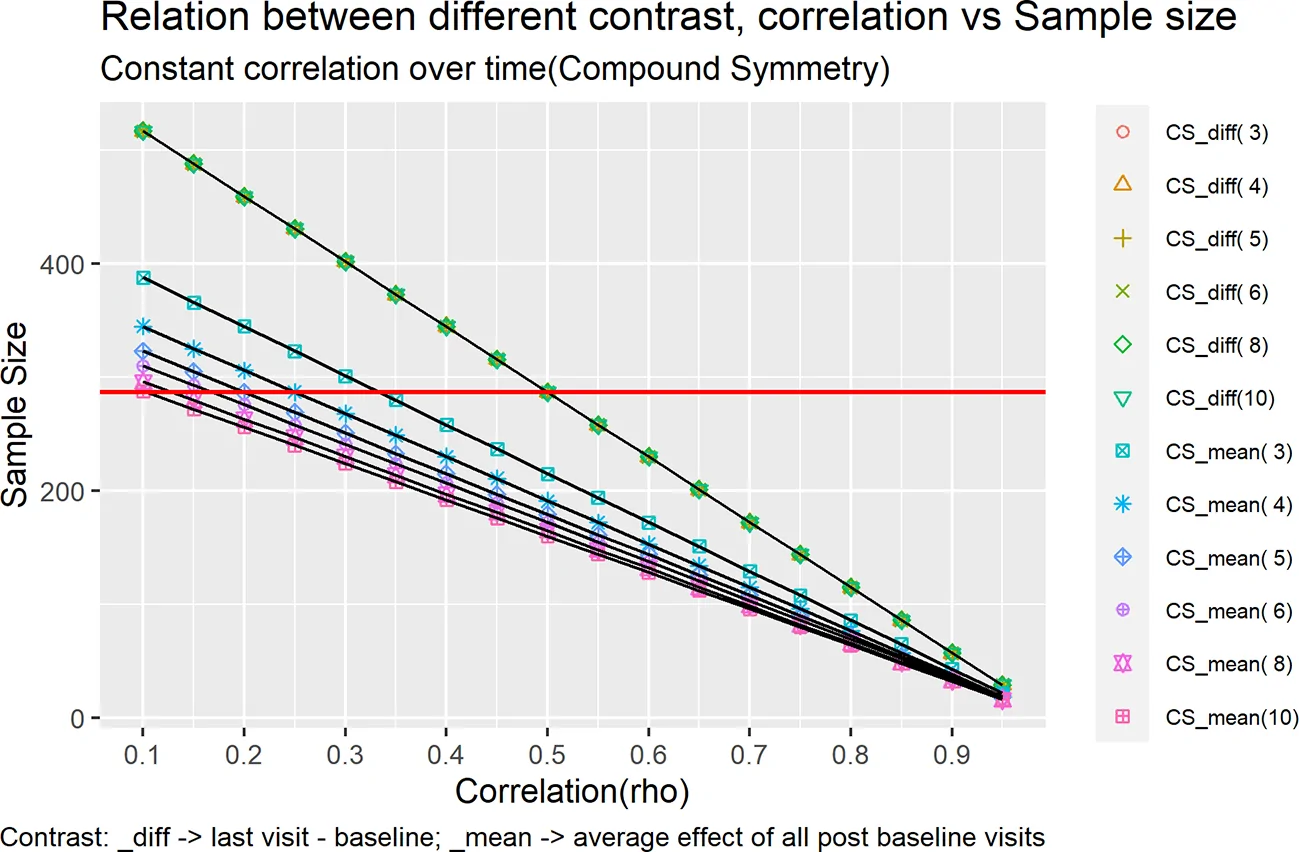
Simulation results with compound symmetry correlation structure.

**Figure 2.**
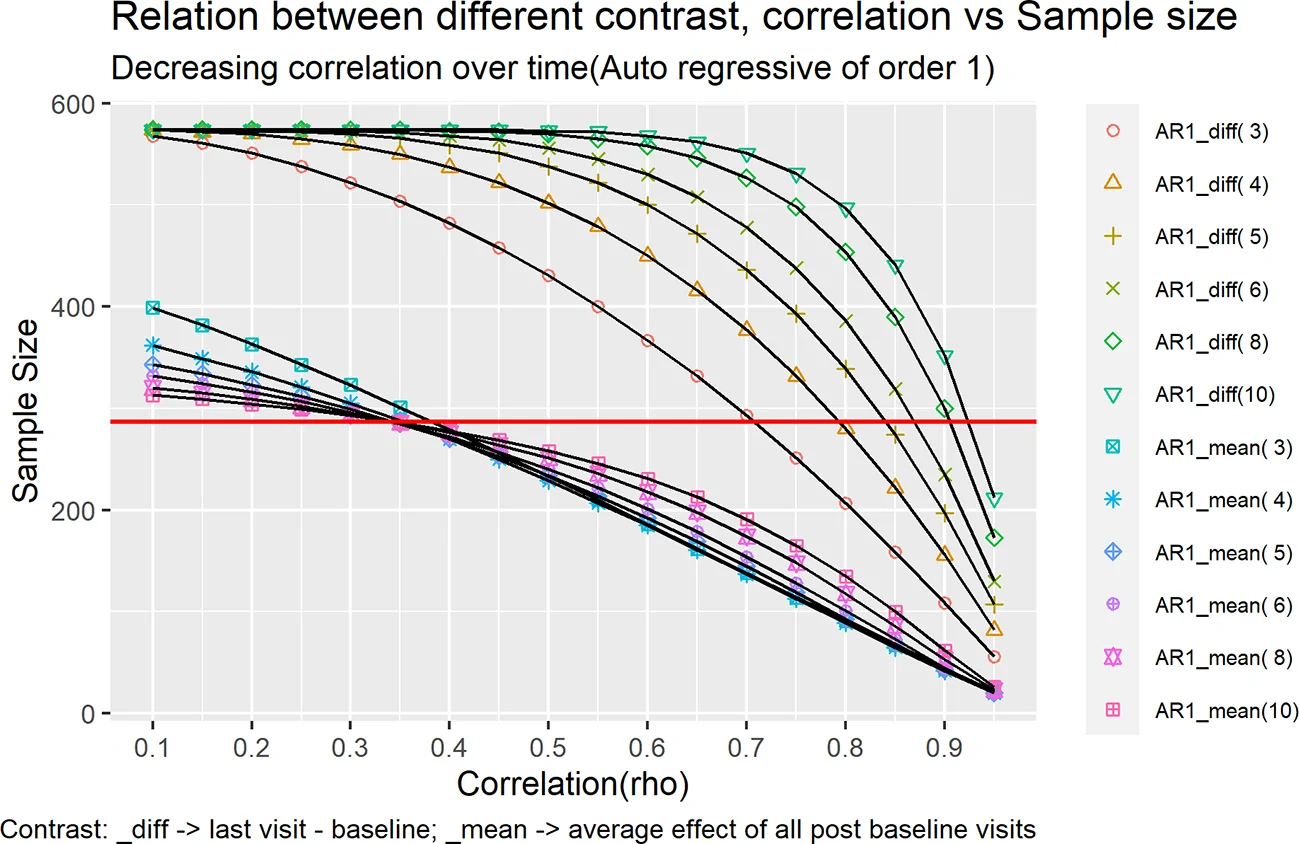
Simulation results with discrete auto regressive of order 1 correlation structure.

Keeping the SD as 3.6 we tried to vary over two different correlation structures:

Compound symmetry (CS)

Compound symmetry just means that all the variances are equal and all the covariances are equal. So, the same variance and covariance are used for all subjects. In compound symmetry the covariances across the subjects and the variances (pooled within the group) of the different repeated measures are homogeneous.

$$\left[\begin{array}{ccccc} \sigma^{2} & \sigma^{2}\rho & \sigma^{2}\rho & \cdots & \sigma^{2}\rho \\ \sigma^{2}\rho & \sigma^{2} & \sigma^{2}\rho & \cdots & \sigma^{2}\rho \\ \sigma^{2}\rho & \sigma^{2}\rho & \sigma^{2} & \cdots & \sigma^{2}\rho \\ \vdots & \ddots & \vdots & \vdots & \vdots \\ \sigma^{2}\rho & \sigma^{2}\rho & \sigma^{2}\rho & \cdots & \sigma^{2} \end{array}\right]$$

Where *σ* ^2^ is the common variance assumed to be similar over time and *ρ* is the assumed correlation. The order of variance covariance matrix will be t × t , where ‘ *t*’ is the number of time points. Generally, *σ* ^2^ and *ρ* are estimated from previous clinical trial data with same molecule which could be phase1, phase2 trials for same indication or pivotal trials with same molecule for different indication.

Discrete Auto regressive of order 1(AR1)

This is the homogeneous variance first-order autoregressive structure. Any two elements that are adjacent have a correlation that is equal to rho ( *ρ*), those separated by a third will have correlation *ρ* ^2^, and so on. rho is restricted such that –1< *ρ* <1.

$$\left[\begin{array}{ccccc} \sigma^{2} & \sigma^{2}\rho & \sigma^{2}\rho^{2} & \cdots & \sigma^{2}\rho^{t-1}\\ \sigma^{2}\rho & \sigma^{2} & \sigma^{2}\rho & \cdots & \sigma^{2}\rho^{t-2}\\ \sigma^{2}\rho^{2} & \sigma^{2}\rho & \sigma^{2} & \cdots & \sigma^{2}\rho^{t-3}\\ \vdots & \ddots & \vdots & \vdots & \vdots \\ \sigma^{2}\rho^{t-1} & \sigma^{2}\rho^{t-2} & \sigma^{2}\rho^{t-3} & \cdots & \sigma^{2} \end{array}\right]$$

Where *σ* ^2^ is the common variance assumed to be similar over time and *ρ* is the assumed correlation. The order of variance covariance matrix will be t × t , where ‘ *t*’ is the number of time points. Generally, *σ* ^2^ and *ρ* are estimated from previous clinical trial data with same molecule which could be phase1, phase2 trials for same indication or pivotal trials with same molecule for different indication.

Also, we considered different scenarios of how we want to analyze the results at the end as different contrasts as described below.

### Contrast of repeated measures

We investigated two types of contrasts.

1. Time-related contrasts i.e., mean over time (mean contrast).

**Rationale:** This contrast is more applicable in the situation where interest lies in observing the mean treatment effect on the subject’s disease condition over the period of time the subject is exposed to a prescribed dosing regimen compared to baseline.
This will be labelled in the legend of Figure 1 as CS_mean( *i*) and in the legend of Figure 2 as AR1_mean( *i*). For example, for five timepoints the contrast would look like $C^{\prime }=$ [-1, ¼, ¼, ¼, ¼].
The mean-over-time contrast with equal weights was chosen to estimate the average treatment effect across follow-up, which is clinically meaningful in chronic disease settings and does not rely on the treatment effect following a strictly linear pattern over time.
2. Mean difference (change at last time point from baseline) (diff contrast).

**Rationale:** This contrast is more applicable in the situation where the interest lies in observing the treatment effect once the subject is exposed to a prescribed dosing regimen and what is the change at the end of the treatment exposure period in the subject’s disease condition. Here the total effect at the end of the treatment course as compared to the baseline is of interest.
This will be labelled in the legend of Figure 1 as CS_diff( *i*) and in the legend of Figure 2 as AR1_diff( *i*). For example, for five timepoints the contrast would look like $C^{\prime }=$ [-1, 0, 0, 0, 1].

The sample size was calculated for correlation ranging from 0.1 – 0.95 with intervals of 0.05 for both the plots Figure 1 and Figure 2, using the formulas outlined in Equation 13.

### CS variance structure with ‘mean over time’ and ‘mean difference’ contrasts

The red horizontal line represents the sample size from the single time point assessment approach.

CS_diff( *i*), CS_mean( *i*) – ‘ *i*’ represents the no. of visits used for sample size calculation, and *i* = 3,4,5,6,8,10.

### AR(1) covariance structure with ‘mean over time’ and ‘mean difference’ contrasts

The red horizontal line represents the sample size from the single time point assessment approach.

AR1_diff( *i*), AR1_mean( *i*) – ‘ *i*’ represents the no. of visits used for sample size calculation, and *i* = 3,4,5,6,8,10.

## Results

### Under CS type of variance covariance structure ( Figure 1)

All the trend lines for mean difference type of contrast overlaps each other. For mean difference type of contrast, the sample size does not change for an increase/decrease in the number of visits. It changes with the correlation i.e., highly correlated (rho > 0.5) timepoints would need less sample size as compared to low correlated timepoints. Also, for correlation = 0.5 the multiple assessment sample size coincides with that of single time point assessment.

However, the sample size does vary when the contrast is set to mean over time. Multiple time point assessments with more timepoints require less sample size as compared to that of multiple time point assessment with less time points for example, the multiple time point assessment with three timepoint requires 86 per arm with correlation 0.8 and the multiple time point assessment with ten timepoints requires 64 per arm. On the same lines the multiple time point assessment with three timepoints requires 258 per arm with correlation 0.4 and the multiple time point assessment with ten timepoints requires 192 per arm. This trend shows that under compound symmetry, the efficiency gain from repeated measurement depends on the within subject correlation. The required sample size decreases as the correlation between timepoints increases. However, under CS, at moderate correlation levels (ρ = 0.5), this gain may be neutral, whereas lower correlations reduce efficiency and increase the required sample size.

### Under AR(1) type of variance covariance structure ( Figure 2)

Under mean over time contrast the multiple time point assessment requires lower sample size as compared to single time point assessment (287 per arm) for correlation greater than 0.35 and the sample size increases as correlation goes below 0.35. For correlation 0.35 the sample size coincides with that of single time point assessment for all the cases except the case of three timepoints which requires slightly higher sample number.

Whereas for mean difference contrast the multiple time point assessment requires lower sample size as compared to single time point assessment (287 per arm) for correlation greater than 0.7 but requires higher sample size for correlation less than 0.7.

The trend changes shape for **mean difference** contrast vs mean over time contrast. Also, at certain point the increase in sample size attenuates for example, in case of mean difference type contrast with ten time points the sample size required does not change when correlation drops below 0.55.

## Discussion

One of the hurdles in considering the longitudinal methodology for sample size calculation is the assumption on the covariance matrix. It is often easy to estimate the variance of single timepoint as compared to estimating the variance-covariance matrix for multiple time points.

The above derivations were done for trial design with parallel group, 1:1 ratio and two arms. If the ratio changes or if we have more than two arms or if the design is crossover, then the effective overall sample size would get effected in both the cases i.e., sample size with single time point as well as sample size with multiple timepoints, but the trend would remain the same as shown above in the figures and the results will still hold good. Similar trends should hold for other variance – covariance structures though they have not been simulated here.

## Conclusion

Sample size changes depending on the analysis type and the data collected. Both the graphs in Figure 1 and Figure 2 in this study reveal that if response is assessed at multiple timepoints and the correlation between the paired observations is high (> 0.6) then one should consider using repeated measures analysis and consequently determine the size of the sample that is based on the multiple time points scenario which results in lower sample size requirement as compared to the sample size derived assuming single timepoint response assessment. This would reduce the cost, resources, and time in conducting the experiment fastening the new drug development. Also, repeated measures analyses will not drop the patients in which they have certain missing data as compared to single point analysis where the patient will be dropped if the response is missing and hence may help in retaining the power.

Under a compound symmetry (CS) covariance structure, the required sample size for the mean over time contrast decreases when information from a greater number of time points is available, reflecting the additional information gained from repeated measurements. In contrast, for the mean diff contrast, which is based on a comparison between two time points only, the required sample size remains unchanged regardless of the total number of additional time points observed.

Under an AR(1) covariance structure, different patterns are observed. For the mean over time contrast, the required sample size increases as more time points are included when the within-subject correlation exceeds *ρ* = 0.35, whereas for *ρ* ≤ 0.35 the required sample size decreases with the inclusion of additional time points. For the mean diff contrast, the required sample size with information on fewer timepoints is lower as compared to the sample size required when we have information available at more timepoints ( Figure 2).

These patterns are not universal but depend on the assumed correlation structure and the magnitude of the mean difference at the chosen time point. Further investigation is warranted to better understand how these relationships vary across alternative covariance structures and effect trajectories.

Sample size derivation using longitudinal design method for studies with multiple assessments can be considered of substantial benefit in cost and time although the challenge of estimating the variance-covariance matrix remains. When within subject correlations are low, averaging treatment effects across multiple time points may increase estimator variance, thereby reducing efficiency relative to a single and well-chosen time point comparison. Also, to be noted that the distance between the timepoints is not taken into consideration by the sample size with multiple timepoints derivation method which could be a topic of further research.

The sample size derivation for multiple timepoint assessment Equation 13 can directly be used by the readers using their treatment effect, variance covariance matrix and the contrast of their choice. To ease this, we have also provided the link to R code under software availability section where inputs for treatment effect, variance covariance matrix and contrast can be given to the function to obtain the sample size required. To explore more on power analysis in longitudinal studies one may refer to the book “Sample Size Calculations for Clustered and Longitudinal Outcomes in Clinical Research”. ^13^ One could also make use of the R packages for different cases of longitudinal power analyses provided under the ‘The R Journal’ article “Power and Sample Size for Longitudinal Models in R”. ^17^

## Software availability

Software available from: The Comprehensive R Archive Network ( https://cran.r-project.org/)

Source code available from: https://github.com/Sarfaraz-Sayyed/Sample-Size-Variation.

Archived source code at time of publication: https://zenodo.org/badge/latestdoi/547747570. ^18^

Archived source code at time of revision: Sample Size Variation (zenodo.org)

License: MIT License.

## Data Availability

Supplementary appendices offering detailed clarification of selected outcomes to support deeper methodological understanding are available on Zenodo: https://doi.org/10.5281/zenodo.18453058. ^19^
