# Supplementary material for: Sample size variation in single-time post-dose assessment vs multi-time post-dose assessment
Source: F1000Res. 2026 Sep 30;11:1550. Originally published 2022 Dec 21. [Version 4] doi: 10.12688/f1000research.124917.4 (PMC13662387; doi:10.12688/f1000research.124917.4)
Supplement: Peer review history

# Reviewer report: Reviewer response for version 4

**DOI:** 10.5256/f1000research.211293.r525738

**Published:** 2026-10-14

**Authors:** Lucas Damiani

**Affiliations:** 1 Brazilian Clinical Research Institute, São Paulo, Brazil

I have reviewed Version 4 of the manuscript and the authors’ responses to my comments. All the issues I had identified have been adequately addressed, and I have no further comments.

Is the rationale for developing the new method (or application) clearly explained?

Yes

Is the description of the method technically sound?

Yes

Are the conclusions about the method and its performance adequately supported by the findings presented in the article?

Partly

If any results are presented, are all the source data underlying the results available to ensure full reproducibility?

Partly

Are sufficient details provided to allow replication of the method development and its use by others?

Yes

Reviewer Expertise:

Biostatistics; Sample Size; Generalized Linear Equations; Bayesian Statistics

I confirm that I have read this submission and believe that I have an appropriate level of expertise to confirm that it is of an acceptable scientific standard.

# Reviewer report: Reviewer response for version 3

**DOI:** 10.5256/f1000research.179717.r510179

**Published:** 2026-08-31

**Authors:** Lucas Damiani

**Affiliations:** 1 Brazilian Clinical Research Institute, São Paulo, Brazil

The paper provides an accessible comparison between sample size calculations based on a single post-baseline assessment and calculations that incorporate multiple post-baseline measurements under simple correlation structures. The practical examples may be useful for applied statisticians.

**Major Comments**

 1. The results (Figure 1 and 2) are being presented before the definitions of CS and AR1, which makes the reading difficult, please present all the definitions used in the simulations before the results.

 2.The first conclusion paragraph appears to imply that repeated measures analyses should generally be preferred. While I largely agree, this argument assumes that single time point analyses are based solely on follow-up measurements. In many studies, however, treatment effects are estimated using ANCOVA adjusted for baseline, and sample size calculations are based as presented in the single time point assessment using estimative from the change from baseline. As a result, the correlation between baseline and follow-up measurements is already accounted for. Moreover, when the standard deviation of the change is unknown, an assumption of 0.5 is commonly used for planning purposes, leading to a scenario similar to the one presented in the manuscript. I therefore encourage the authors to discuss this distinction in the Conclusions, maybe also including examples like those in the Introduction.

 3. The third conclusion paragraph states that "For the mean diff contrast, the required sample size with information on fewer timepoints is lower as compared to the sample size required when we have information available at more timepoints". This conclusion appears somewhat counterintuitive for _diff when considered alongside the scenario presented in Table 1, where the primary comparison is performed at the 36-month time point regardless of the number of intermediate assessments collected.

 The observed result is mainly driven by the AR (1) correlation structure, under which the correlation between baseline and the final visit decreases as the number of time intervals increases (\rho^{t-1}). While this explains the numerical findings, it does not parallel the practical scenario motivating the manuscript. Indeed, one could interpret the results as suggesting that fewer measurements should be collected in order to reduce the required sample size, which is clearly not a desirable or realistic conclusion.

**Minor Comments**

 1. Indicate that the proof of "for correlation = 0.5 the multiple assessment sample size coincides with that of single time point assessment." is in the Appendix.

 2. Figures 1 and 2 should include a footnote indicating what the red line is. (the single time point estimative at...)

Is the rationale for developing the new method (or application) clearly explained?

Yes

Is the description of the method technically sound?

Yes

Are the conclusions about the method and its performance adequately supported by the findings presented in the article?

Partly

If any results are presented, are all the source data underlying the results available to ensure full reproducibility?

Partly

Are sufficient details provided to allow replication of the method development and its use by others?

Yes

Reviewer Expertise:

Biostatistics; sample size

I confirm that I have read this submission and believe that I have an appropriate level of expertise to state that I do not consider it to be of an acceptable scientific standard, for reasons outlined above.

# Response

**Published:** 2026-09-23

**Authors:** Sarfaraz Sayyed

**Affiliations:** Manipal University, India

**Response to Major Comment 1:** We thank the reviewer for this valuable observation. We agree that the original presentation required readers to interpret the simulation results before all covariance structures and contrast definitions had been introduced. To improve clarity, we have revised the manuscript by moving the descriptions of the Compound Symmetry and Autoregressive of order 1 covariance structures, along with the definitions and rationale for the "mean over time" and "mean difference" contrasts, to the Methods section before the presentation of Figures 1 and 2 and the Results section. This reorganization provides the necessary context before the simulation outcomes are discussed and improves the overall readability of the manuscript.

**Response to Major Comment 2:** We thank the reviewer for this important observation. We agree that the original conclusion could be interpreted as favouring repeated-measures approaches over single-endpoint analyses. We have therefore revised both the Discussion and Conclusion sections to clarify that baseline-adjusted approaches such as ANCOVA and change-from-baseline analyses already account for the correlation between baseline and follow-up measurements. We further clarified that the primary contribution of this work is to illustrate the potential efficiency gains achieved when multiple post-baseline assessments are incorporated into sample size determination, compared with approaches relying on a single endpoint assessment. We also linked this interpretation to the examples discussed in the Introduction.

**Response to Major Comment 3**: We thank the reviewer for this important observation. We agree that the original wording could be misinterpreted as suggesting that fewer measurements should be collected in order to reduce sample size. This was not our intention. We have revised the Discussion and Conclusion sections to clarify that the observed pattern is primarily driven by the AR(1) covariance structure assumed in the illustrative examples, whereby the correlation between baseline and the final assessment decreases as additional intervals are introduced. We now explicitly state that this finding should not be interpreted as a recommendation to reduce the number of measurements, but rather as an illustration of the sensitivity of longitudinal sample size calculations to the assumed covariance structure. We further emphasize that assessment schedules should continue to be guided by the scientific and clinical objectives of the study.

**Response to Minor Comment 1: **We thank the reviewer for this suggestion. To improve clarity, we have revised the relevant sentence in the Results section to explicitly direct readers to the supplementary material. The manuscript now states that the mathematical derivation/proof demonstrating why the multiple-assessment sample size coincides with the single-time-point sample size when the correlation equals 0.5 is provided in Appendix 1 of the Supplementary Appendices.

**Response to Minor Comment 2:** We thank the reviewer for this helpful suggestion. To improve clarity and interpretation of the figures, we have added footnotes to Figures 1 and 2 explicitly identifying the red line as the sample size estimate obtained under the single time point assessment scenario. The figure captions/footnotes have been updated accordingly.

**Response to justify "source data underlying the results available to ensure full reproducibility":** Thank you for your feedback. No empirical data were analyzed in this study. All results presented in the manuscript are figures generated from the assumptions and parameter values explicitly described in the Methods section. The R code used to generate all figures and plots is publicly available in the GitHub repository. Therefore, all information required to reproduce the presented results, including the analytical code and model assumptions, has been provided. We would appreciate any clarification regarding the aspects of reproducibility that you feel are not yet fully addressed, so that we can further improve the manuscript.

**Response to "conclusions about the method and its performance being adequately supported by the findings presented in the article"**: Thank you for your feedback. The concerns underlying this comment have been addressed through the revisions made in response to the second and third major comments. These changes strengthen the link between the presented findings and the conclusions regarding the proposed method and its performance.

# Reviewer report: Reviewer response for version 2

**DOI:** 10.5256/f1000research.170509.r333804

**Published:** 2024-11-21

**Authors:** Simon Vandekar

**Affiliations:** 1 Vanderbilt University Medical Center, Tennessee, USA

The paper doesn’t reference the huge body of literature on power analysis in longitudinal studies. There are 14 references and only of them is a classical text on longitudinal data analysis (Diggle et al). For example, the book by Ahn, Heo, Zhang “Sample Size Calculations for Clustered and Longitudinal Outcomes in Clinical Research.” There are numerous R package for longitudinal power analyses, but these were not discussed (e.g. https://journal.r-project.org/articles/RJ-2022-022/). The results and topic are not novel and overlook critical references that have studied the topic. Given the very general topic, I would have expected more references to the existing literature.

 There need to be more references for the mathematics in the paper, such as “Method for Sample size with single time point assessment analysis.” Grammar in the paper needs to be reviewed. There are some notation errors, e.g. in equations (8) and (9) Var(\Gamma) doesn’t make sense because Gamma is defined as a parameter (so it has zero variance).

Is the rationale for developing the new method (or application) clearly explained?

Yes

Is the description of the method technically sound?

Partly

Are the conclusions about the method and its performance adequately supported by the findings presented in the article?

Partly

If any results are presented, are all the source data underlying the results available to ensure full reproducibility?

Yes

Are sufficient details provided to allow replication of the method development and its use by others?

Yes

Reviewer Expertise:

Biostatistics

I confirm that I have read this submission and believe that I have an appropriate level of expertise to state that I do not consider it to be of an acceptable scientific standard, for reasons outlined above.

## References

[1] : Sample Size Calculations for Clustered and Longitudinal Outcomes in Clinical Research.2014; 10.1201/b17822 10.1201/b17822

# Response

**Published:** 2026-01-18

**Authors:** Sarfaraz Sayyed

**Affiliations:** Manipal University, India

**Response to Comment 1**

 We thank the reviewer for this important and thorough comment and fully agree that there is a substantial body of literature on power and sample size calculation for longitudinal studies. Our intent was not to provide a comprehensive review or to introduce new theoretical developments in this well‑established area. Rather, the aim of the present paper is to provide a focused and accessible comparison between sample size calculations based on a single post‑baseline assessment and those incorporating multiple post‑baseline time points, under commonly used correlation structures.

 To better position our work within the existing literature, we have expanded the related work section to explicitly acknowledge classical and contemporary references on longitudinal power analysis, including Diggle et al. and the monograph by Ahn, Heo, and Zhang. We have also added references to existing R packages for longitudinal power analysis and clarified how our approach complements these tools by highlighting specific design trade‑offs in simple, interpretable settings.

 We hope that these revisions more clearly situate the contribution of this paper as illustrative and comparative rather than novel in a methodological sense, while providing practical insights for applied researchers.

**Response to Comment 2**

 We thank the reviewer for this detailed comment. In the revised manuscript, we have added appropriate references for the single‑time‑point sample size calculation methodology. We have also carefully reviewed the manuscript for grammatical issues and corrected several notation inconsistencies. In particular, we revised Equations (8) and (9) to avoid expressions such as Var( ), as  represents a fixed parameter, and reformulated these expressions using appropriate sample‑based quantities. We believe these revisions improve both the mathematical clarity and presentation of the manuscript.

# Reviewer report: Reviewer response for version 2

**DOI:** 10.5256/f1000research.170509.r320982

**Published:** 2024-10-23

**Authors:** Kiranmoy Das

**Affiliations:** 1 Indian Statistical Institute, Kolkata, West Bengal, India

I have seen the revised version of the paper and the author's comments.

 I am quite satisfied and I have no additional comments.

 I recommend the acceptance of the manuscript

Is the rationale for developing the new method (or application) clearly explained?

No

Is the description of the method technically sound?

Yes

Are the conclusions about the method and its performance adequately supported by the findings presented in the article?

Yes

If any results are presented, are all the source data underlying the results available to ensure full reproducibility?

Yes

Are sufficient details provided to allow replication of the method development and its use by others?

No

Reviewer Expertise:

Biostatistics, Longitudinal data analysis, Bayesian modeling

I confirm that I have read this submission and believe that I have an appropriate level of expertise to confirm that it is of an acceptable scientific standard.

# Reviewer report: Reviewer response for version 2

**DOI:** 10.5256/f1000research.170509.r320983

**Published:** 2024-09-28

**Authors:** Ronald Geskus

**Affiliations:** 1 Oxford University, Oxford, England, UK

The article still contains several typos, sentences that abruptly end and capital letters that are misplaced. I suggest the authors do a careful check of typos. Also, I still found a couple of errors, as well as a few statements that are not clear to me.

 1. "It affects the estimator’s precision, which is a metric that is important for the chosen target population of all professional athletes". Precision is a characteristic of the data. Explain why precision is an important metric for the target population.

 2. The authors found only two papers (references 3 and 4) in their literature review on longitudinal studies? There must be many more.

 3. Spiegelma  -> Spiegelman

 4. Page 4: Make clear that the normal distribution of the sample mean is an approximation, which becomes more accurate with increasing sample size (based on the central limit theorem)

 5. The authors replied: "Comment 4: We fully agree with you, this was a generic equation to show we are looking positive change in response and then further with the examples we have assumed for certain fixed delta how the sample size varies. But have modified it to avoid confusion." I doubt whether "H_a  is true" and formula (2) avoid confusion. To my opinion, it is clearer to change "H_a is true" and "\delta > 0" by a specific value for \delta.

 6. Page 5: H_a -> H_0 for null hypothesis, and \psi_c=C^t \Lambda, with "t" meaning transpose.

 7. Formula (8) is not a test statistic, \psi_c is a property of the population not based on random variables. Also, \Lambda is a population property, not determined by random variables.

 8. covar(Y_2-X_2,Y_1-X_1)=covar(Y_2,Y_1)+covar(X_2,X_1). I don't see where the value 4 comes from in (9).

 9. I still find the section "Calculation of sample size" not very clearly phrased. Specify what is meant with "the last time point" (later it is written to be 3 years). What time trend does it imply? Replace "allowing" by "choosing"

 10. Explain how the sample size of 287 is obtained. How large is the assumed difference under the null hypothesis, i.e. what is the chosen time point?

 11. The rationale for the mean over time contrast chosen is still not clear to me. Why would one choose 1/4 at later time points, given that a linear trend is assumed?

 12. Can the authors explain i) why the sample size for correlation=0.5 (compound symmetry) coincides with single time point assessment and ii) why the required sample size is larger if correlation<0.5?

 13. I do not understand why with low correlation the single time point assessment performs best. Please explain.

 14. I do not understand why the sample size for AR(1) mean over time contrast increases with increasing number of time points included if correlation > 0.35. Please explain.

 15. Page 11: "Whereas for the mean diff contrast the sample size required with fewer visit is lower as compared to the sample size required with higher number of visits". It's not the number of visits that matters (it is always based on two time points only), but the distance between the visits. The required sample size for AR(1) mean difference contrast increases if the second time point is further away from the baseline. I don't think this is a general property. It will depend on the combination of serial correlation and difference in mean at the chosen time point. This needs further investigation.

 16. The authors give a detailed introduction for readers who do not have a statistical background. It would be very useful to provide some guidance how the results can directly be used by them.

Is the rationale for developing the new method (or application) clearly explained?

No

Is the description of the method technically sound?

Yes

Are the conclusions about the method and its performance adequately supported by the findings presented in the article?

Partly

If any results are presented, are all the source data underlying the results available to ensure full reproducibility?

Yes

Are sufficient details provided to allow replication of the method development and its use by others?

Yes

Reviewer Expertise:

biostatistics

I confirm that I have read this submission and believe that I have an appropriate level of expertise to state that I do not consider it to be of an acceptable scientific standard, for reasons outlined above.

# Response

**Published:** 2026-01-18

**Authors:** Sarfaraz Sayyed

**Affiliations:** Manipal University, India

**Response to General comment**

 We thank the reviewer for the comment. In the revised version, we have carefully corrected typographical errors, incomplete sentences, and improperly placed capital letters, and clarified statements that were previously unclear.

**Response to Comment 1**

 We thank the reviewer for this important clarification. In the revised manuscript, we have expanded the Introduction to explicitly explain why estimator precision is a critical metric for the target population of professional athletes. Specifically, we emphasize that professional athletes exhibit high inter- and intra-individual variability, and that precise estimation is essential for accurate performance assessment, monitoring, and interpretation of small but practically meaningful effects. We believe this added explanation clarifies the relevance of precision for the chosen population.

**Response to Comment 2**

 We thank the reviewer for this comment. We agree that there are numerous longitudinal studies in the literature. However, our intention was not to provide a comprehensive review of longitudinal research, but rather to illustrate that even in well‑established and prestigious journals, longitudinal studies often provide limited detail regarding sample size calculation. We have now clarified this scope in the manuscript to avoid any misunderstanding and to emphasize that references 3 and 4 were cited as illustrative examples rather than as an exhaustive review of the literature.

**Response to Comment 3**

 We thank the reviewer for pointing this out. The spelling has been corrected in the revised version of the manuscript

**Response to Comment 4**

 We thank the reviewer for this clarification. We have now explicitly stated that the normal distribution of the sample mean is an approximation that becomes increasingly accurate with larger sample sizes, as justified by the central limit theorem. This clarification has been added to the ‘Notation and framework’ section in the revised manuscript.

**Response to Comment 5**

 We thank the reviewer for this helpful suggestion. To improve clarity, we have revised the hypotheses to explicitly state “ ” and “  , where  *,*  and *δ* represents the expected difference. This definition clarifies the role of δ in the sample size derivation and avoids ambiguous statements. The corresponding revision has been made in the Methods section and aligns with Equation (2) accordingly.

**Response to Comment 6**

 We thank the reviewer for pointing this out. The null and alternative hypothesis notation and the definition of the transpose operator have been corrected in the revised manuscript.

**Response to Comment 7**

 We thank the reviewer for this important clarification. We agree that ψ_c and Λ, as population parameters, cannot constitute a test statistic. In the revised manuscript, we have therefore replaced these quantities with their corresponding sample estimates and revised the notation accordingly. As a result, Formula (8) is now explicitly defined as a function of sample‑based random variables, ensuring that it represents a valid test statistic.

**Response to Comment 8**

 We thank the reviewer for carefully examining the derivation. We agree that the covariance expansion was incorrectly stated and that the origin of the value 4 in Equation (9) was not justified. In the revised manuscript, we have corrected the covariance expression by explicitly accounting for all relevant covariance terms and have revised Equation (9) accordingly. The incorrect constant has been removed, and the derivation is now consistent with standard covariance identities.

**Response to Comment 9**

 We thank the reviewer for these constructive suggestions to improve clarity. In the revised manuscript, we have explicitly defined and clarified the assumed time trend in the sample size calculation. We have also revised the wording as suggested, replacing “allowing” with “choosing.” In addition, we have added a summary table to clearly indicate the selected time points under each scenario.

**Response to Comment 10**

 We thank the reviewer for this important query. In the revised manuscript, we have explicitly detailed how the sample size of 287 was derived, including the expected difference under the alternative hypothesis, the corresponding null hypothesis, and the selected time point used in the sample size calculation, thereby making the derivation and underlying assumptions transparent and reproducible.

**Response to Comment 11**

 We thank the reviewer for this insightful comment. Our objective in choosing a mean‑over‑time contrast was to target the average treatment effect across the follow‑up period rather than the effect at a single time point. Accordingly, equal weights (1/4 at each post‑baseline time point) were used to reflect this estimand.

 Although a linear time trend is assumed for the purpose of modeling and sample size calculation, the choice of an average contrast does not require linearity of the treatment effect over time. This approach is particularly relevant in chronic disease settings, where sustained control of biomarkers over time is often of greater clinical interest than the maximum effect at a specific visit, and it also provides robustness to missing data due to loss to follow‑up.

 We have clarified this rationale in the revised manuscript to better explain the choice of the equal‑weight contrast and its relationship to the assumed time trend.

**Response to Comment 12**

 We thank the reviewer for this insightful question. Under a compound symmetry correlation structure, when the within‑subject correlation is 0.5, the variance reduction gained from repeated measurements is exactly offset by their correlation, resulting in a required sample size equivalent to that of a single‑time‑point assessment. When the correlation is lower than 0.5, repeated observations provide less shared information across time points, leading to a reduced efficiency and consequently a larger required sample size.

 To provide a detailed mathematical justification of these results, we have added two appendices that explicitly derive the variance expressions and resulting sample size formulas under different correlation assumptions.

**Response to Comment 13**

 We thank the reviewer for this important question. When within‑subject correlation is low, repeated measurements provide relatively little shared information about the underlying treatment effect, as observations across time behave nearly independently. For contrasts that average treatment effects over time, this can lead to increased variability because the additional measurements contribute more noise than signal.

 In contrast, a single time‑point assessment, particularly at a clinically relevant follow‑up time point may yield a more efficient estimator under low-correlation settings, as it avoids the accumulation of variability from weakly correlated repeated measures. This explains why, in our framework, the single‑time‑point assessment can require a smaller sample size when the correlation is low. Appendix added can also give a mathematical glimpse.

**Response to Comment 14**

 We thank the reviewer for this insightful question. Under an AR(1) correlation structure, increasing the number of time points does not necessarily lead to increased efficiency when within‑subject correlation is moderate to high. As additional time points are included, the covariance between observations accumulates, and beyond a certain correlation threshold, the variance component associated with the mean‑over‑time contrast ( ) increases rather than decreasing.

 This occurs because strongly correlated repeated measurements contribute limited new information while increasing the overall covariance contribution to the contrast variance. As a result, when the correlation exceeds approximately 0.35 in our setting, adding more time points inflates the variance of the mean‑over‑time estimator, leading to a higher required sample size. To clarify this behavior, we have added an appendix that explicitly compares variance components across different correlation levels and side by side layout for 4 timepoints and 10 timepoints case.

**Response to Comment 15**

 We thank the reviewer for this important clarification. We agree that, for the mean difference contrast, the comparison is based on two time points (baseline and a post‑baseline visit), and that it is the temporal distance between these time points, rather than the total number of visits that determines the correlation and variance under an AR(1) structure. We have revised the manuscript to clarify this wording and avoid any implication that the total number of visits alone drives the sample size.

 We also agree that the observed increase in required sample size when the post‑baseline assessment is further from baseline is not a general property but depends on the joint behaviour of the serial correlation and the mean difference at the chosen time point. This dependence is now acknowledged more explicitly in the revised text and reiterated in the conclusion.

**Response to Comment 16**

 We thank the reviewer for this helpful suggestion. To facilitate direct use by readers without a statistical background, the manuscript provides explicit formulas for both single and multiple time point sample size calculations, along with openly available R code (see Software Availability). Users can obtain required sample sizes by specifying interpretable inputs such as the anticipated treatment effect, the assumed variance-covariance structure, and the contrast of interest. In addition, we have added a worked example in the Appendices demonstrating how these inputs are selected and applied in a realistic clinical trial setting.

# Reviewer report: Reviewer response for version 1

**DOI:** 10.5256/f1000research.137160.r294678

**Published:** 2024-07-10

**Authors:** Ronald Geskus

**Affiliations:** 1 Oxford University, Oxford, England, UK

The authors describe some formulas for sample size calculations with longitudinal data with a continuous outcome. The authors only consider one specific approach, namely generalized least squares and comparing changes from baseline. This latter approach is discouraged, see e.g. https://www.bmj.com/content/323/7321/1123.

 More general methods to perform sample size and power calculations with longitudinal data have been described and implemented in software (e.g. https://journal.r-project.org/articles/RJ-2022-022/).

 Also, there are some descriptions that need further attention.

 1. The introduction describes the basic principles of hypothesis testing. This can be assumed known and therefore I suggest to leave it out of the paper.

 2. Page 3: The authors describe sampling error as the uncertainty arising from: "the limitation to examine every situation, the absolute precision of the effect of protein shake on the athlete's performance would be hard to measure." I am not sure whether I understand what is written here, but I think it is not correct.

 3. Page 3: Sampling error decreases with increasing sample size, but it is not true that " there comes a point where increasing the sample size has no further effect on the sampling error".

 4. Formula (2): power is typically calculated for a specific choice of the effect size, not for all delta>0 at once.

 5. Page 7: "Appropriate sample size was calculated for multi-time and single time cases with different scenarios to achieve an overall mean treatment difference (0.9 points)." What is meant with mean difference in the situation with more than 2 time points? The difference could vary per time point but still give a mean difference of 0.9.

 The last sentence of that paragraph is irrelevant information.

 6. How was \sigma_{ij} chosen?

 7. Page 8: make more explicit that a discrete AR(1) is assumed. There also exitsts a continuous AR(1)

 8. Page 9: The authors write: "For correlation = 0.1 to 0.35 the sample size coincides with that of single time point assessment." I don't see this in the figure

 9. Page 9: I don't understand the rationale "effect remains only for some time and then the disease condition reverses back' and "each dose reduces the disease severity and the over the course it is totally removed from the patient’s body" for the two contrasts.

 10. Explain why the sample size goes down to zero if the correlation approaches 1. A correlation of 1 does not make sense. It implies that the second measurement is completely determined by the first, and the intervention cannot have any impact.

 11. Legends Fig 1 and 2 have been mixed up.

Is the rationale for developing the new method (or application) clearly explained?

No

Is the description of the method technically sound?

Yes

Are the conclusions about the method and its performance adequately supported by the findings presented in the article?

Partly

If any results are presented, are all the source data underlying the results available to ensure full reproducibility?

Yes

Are sufficient details provided to allow replication of the method development and its use by others?

Yes

Reviewer Expertise:

Biostatistics

I confirm that I have read this submission and believe that I have an appropriate level of expertise to state that I do not consider it to be of an acceptable scientific standard, for reasons outlined above.

# Response

**Published:** 2024-08-18

**Authors:** Sarfaraz Sayyed

**Affiliations:** Manipal University, India

Thanks Dr. Ronald Geskus for your review and advice.

 Please find below our responses.

 - Regarding analyzing change from baseline, we completely agree with you. Our intention was just to propose a framework, but we have modified it now to make it more precise.

 - Regarding methods to perform sample size calculation, we agree with you, but our intention here is to highlight using simple methods to the clinical researchers, the difference in samples required when we have longitudinal information available under 2 different correlation structures.

 Comment 1: We kept this as this would be helpful to new researchers not having statistical background.

 Comment 2: We have modified it for better understanding.

 Comment 3: We have modified and added a note to have more clarity.

 Comment 4: We fully agree with you, this was a generic equation to show we are looking positive change in response and then further with the examples we have assumed for certain fixed delta how the sample size varies. But have modified it to avoid confusion.

 Comment 5: We have modified the sentence for better clarity and also removed the last sentence of the paragraph.

 Comment 6: Sigma was taken from a trial data (reference given) and was assumed to be same over all the time periods.

 Comment 7: Thanks for bringing this up, we have updated this in the manuscript now.

 Comment 8: This was wrongly written under figure 1, it was supposed to be under figure 2 conclusion. we have made the necessary changes now.

 Comment 9: The first rationale refers to chronic diseases where the disease can usually be controlled but not cured. So here we are trying to see that the average effect from the treatment is the one which is expected to control the disease. Have changed the wordings for better understanding. The second rationale is for infectious diseases where with the right treatment regimen the disease will be cured. Have now tried to put in better words.

 Comment 10: Agree with your comment on both the extreme cases that a correlation of 1 would imply all the effect is due to intervention not leaving any space for error and also that correlation 0 would mean no effect from the intervention. We have now revised the plots to start from 0.05 till 0.9

 Comment 11: Have made the necessary changes.

 - Regarding clarifying the rationale, basically in clinical trials the use of all time point data is not generally used. The effect at intermittent time points and the covariance structure between time points would be helpful which otherwise not taken into consideration results in large sample size.

 - Regarding conclusion on the methods, we have updated the conclusion to have more insights, how taking into correlation would benefit and also how the effects diminish even if too many timepoints are added.

# Reviewer report: Reviewer response for version 1

**DOI:** 10.5256/f1000research.137160.r273771

**Published:** 2024-05-30

**Authors:** Kiranmoy Das

**Affiliations:** 1 Indian Statistical Institute, Kolkata, West Bengal, India

Reviewer’s comments on the manuscript “Sample-size variation in single-time post-dose assessment vs multi-time post-dose assessment”

 The manuscript attempts to address a very important and interesting problem. However, the presentation is not quite clear, there are lots of typos and grammar issues here and there. The methodology developed in the manuscript can be improved. My comments are given below. After addressing these comments and after a substantial revision, I can make some positive comment on the manuscript.

 1. Introduction: Please provide some good motivation and nice description of the problem. There are several typos. Please avoid the phrase For e.g., this should be For example, and also “The size of the sample and the effect size are major factors which affects”, this should be like “The sample size and effect size are major factors that affect”.

 2. Methods: Equation (1), you are assuming here that the variance sigma^2 is known. Please specify it, and also how appropriate this assumption is in real application?

 3. For the longitudinal studies, your method depends on the auto-correlation \rho. You need to estimate \rho before calculating the sample size. Please clarify that and also discuss how to estimate \rho. Maybe you run a pilot trial and get an estimate?

 4. Both the figures 1 and 2 focus on how the sample size varies with correlation \rho. For a longitudinal trial, it maybe more important to know how it covaries with the number of time points. Correlation is something that cannot be adjusted, but the number of time points can be adjusted as per the requirement. I think this issue has been ignored in the manuscript.

Is the rationale for developing the new method (or application) clearly explained?

No

Is the description of the method technically sound?

Yes

Are the conclusions about the method and its performance adequately supported by the findings presented in the article?

Yes

If any results are presented, are all the source data underlying the results available to ensure full reproducibility?

Yes

Are sufficient details provided to allow replication of the method development and its use by others?

No

Reviewer Expertise:

Biostatistics, Longitudinal data analysis, Bayesian modeling

I confirm that I have read this submission and believe that I have an appropriate level of expertise to state that I do not consider it to be of an acceptable scientific standard, for reasons outlined above.

# Response

**Published:** 2024-08-18

**Authors:** Sarfaraz Sayyed

**Affiliations:** Manipal University, India

Thanks Dr. Kiranmoy Das for your review and advice.

 Please see our response below.

 Comment 1: We have updated the introduction section.

 Comment 2: Justification now added above equation no. 1.

 Comment 3: An explanation of rho estimation is now added.

 Comment 4: We have added a paragraph under the conclusion section to address this.

 We have added a few lines in the Introduction section to clarify the rationale.

 Regarding details for replication of the method, details on assumptions are specified in the section “Calculation of sample size” in the manuscript. The software availability section provides the link to the related R program residing in Github.
